# Nanoparticles for Stem Cell Tracking and the Potential Treatment of Cardiovascular Diseases

**DOI:** 10.3389/fcell.2021.662406

**Published:** 2021-07-02

**Authors:** Huihua Huang, Xuejun Du, Zhiguo He, Zifeng Yan, Wei Han

**Affiliations:** ^1^Emergency Department, Shenzhen University General Hospital, Shenzhen University, Shenzhen, China; ^2^Guangdong Key Laboratory for Biomedical Measurements and Ultrasound Imaging, School of Biomedical Engineering, Shenzhen University, Health Science Center, Shenzhen, China; ^3^Advanced Materials Institute, Graduate School at Shenzhen, Tsinghua University, Shenzhen, China

**Keywords:** nanoparticles, stem cell therapy, mesenchymal stem cell, cardiovascular diseases, imaging technique

## Abstract

Stem cell-based therapies have been shown potential in regenerative medicine. In these cells, mesenchymal stem cells (MSCs) have the ability of self-renewal and being differentiated into different types of cells, such as cardiovascular cells. Moreover, MSCs have low immunogenicity and immunomodulatory properties, and can protect the myocardium, which are ideal qualities for cardiovascular repair. Transplanting mesenchymal stem cells has demonstrated improved outcomes for treating cardiovascular diseases in preclinical trials. However, there still are some challenges, such as their low rate of migration to the ischemic myocardium, low tissue retention, and low survival rate after the transplantation. To solve these problems, an ideal method should be developed to precisely and quantitatively monitor the viability of the transplanted cells *in vivo* for providing the guidance of clinical translation. Cell imaging is an ideal method, but requires a suitable contrast agent to label and track the cells. This article reviews the uses of nanoparticles as contrast agents for tracking MSCs and the challenges of clinical use of MSCs in the potential treatment of cardiovascular diseases.

## Introduction

In the medical world, stem cell-based therapies have emerged and expanded as a potentially promising therapeutic treatment (Heslop et al., 2015). In such a treatment process, stem cells, possessing properties of self-renewal and potency, play a significant role, and under appropriate conditions, these cells can be differentiated into a variety of cells (Srivastava and Ivey, 2006; Maumus et al., 2011; Hao et al., 2014; Aly, 2020). Compared with direct transplantation of tissue or organ for patients, stem cell-based therapies are more promising and potential because these cells can be feasibly and readily obtained avoiding donor shortage and immune incompatibility (Diekman and Guilak, 2013; Lilly et al., 2016). In general, stem cells can be divided into two major types, embryonic stem cells (ESCs) and adult stem cells (ASCs) (Lv et al., 2014). The transplantation of ESCs derived from the early embryo has been demonstrated to be effective in the treatment of many diseases, due to their being highly undifferentiated cells and having the ability of evolving into all tissues and organs (Krebsbach and Robey, 2002; Hao et al., 2009). However, the research for ESCs is controversial, being difficult to accepted by common people (Li et al., 2010). Compared with ESCs, ASCs, which originated from tissues, are easily accepted and have been widely applied in preclinical and clinical trials, but leads to low capacity of differentiation (Young and Black, 2004; Ye et al., 2006; Alison and Islam, 2009). For example, the hematopoietic stem cells (bone marrow), originated from ASCs in the blood, can differentiate into various types of mature blood cells, and their transplantation has been used to treat all kinds of blood diseases (Carella et al., 2000; Schmitz et al., 2002; Mikkola and Orkin, 2006; DiPersio et al., 2009; Jaiswal et al., 2009). Neural stem cells, possessing the ability of differentiating into neurons and astroglia, are also derived from ASCs, and their transplantation may treat diseases of the nervous systems (Temple, 2001; Lee et al., 2005, 2009; Andres et al., 2008; Stenudd et al., 2015). In 1970, Friedenstein discovered a rare type of stromal cells in human bone marrow that are now known as mesenchymal stem cells (MSCs) (Friedenstein et al., 1970). Although MSCs have similar abilities to ASCs, there is still a lot of debate about their origin. Moreover, some researchers claimed that the name of MSCs should be changed to medicinal signaling cells in order to accurately reflect the fact that these cells play the role of constructing new tissues (Caplan, 2017). At present, MSCs are found in many tissues beyond the bone marrow, including adipose tissue, lung tissue, synovial membrane, endometrium, and peripheral blood. In different tissues, MSCs exhibit differences in immunophenotype, differentiation potential, and immunomodulatory. The researches show that the transplantation of MSCs is an effectively promising and potentially therapeutic treatment for various diseases including wound healing, ischemic encephalopathy, and ischemic heart disease (Grauss et al., 2007; Wu et al., 2007; Uccelli et al., 2008; Kim et al., 2015a; Xie et al., 2016).

To date, stem cell-based therapies have been intensively employed in the treatment of wounds, blood and cardiovascular diseases, cartilage defect, diabetes, etc. (Goradel et al., 2018; Vagnozzi et al., 2020). However, there are still much to be further explored for stem cell-based therapies, in order to identify therapeutic efficacy and further dosage (Yi et al., 2017). For these purposes, it is necessary to track stem cells *in vitro* and *in vivo* with high temporal and spatial resolution in a real-time manner during a prolonged period, which involves monitoring stem cells’ behavior *in vivo*, identifying if they are capable of surviving, integrating with the host tissue, undertaking the desired cellular differentiation, and finally unveiling the responses and behaviors of transplanted cells upon exposure to various diseased environments.

## The Means of Tracking Stem Cells and the Importance of Nanoparticles

For tracking stem cells, non-invasive cell imaging is currently a very hot field of research (Kircher et al., 2011). Compared with traditional histopathological techniques, non-invasive cell imaging can monitor the behaviors of the transplanted cells over time *in vivo* for further treatment offering detailed information, thus, showing more efficient therapeutic effects (Cahill et al., 2004; Walczak and Bulte, 2007; Bulte, 2017; Meir and Popovtzer, 2018; Peng et al., 2018). Only by using non-invasive means can the viability of the transplanted cells be estimated in the clinical setting, showing potential prospects (Shang et al., 2017; Tkaczyk, 2017). Non-invasive cell tracking can be divided into direct and indirect labeling techniques. Compared with direct labeling, which can directly label cells, possessing easy operation but being unable to track daughter cells due to fast signal decay, indirect labeling obtains the ability of labeling cells by implanting an indicator into cells (Li et al., 2008; Kraitchman and Bulte, 2009; Kim et al., 2016a,b). Although indirect labeling is complex and expensive, it is more effective and stable for tracking the differentiation of the transplanted cells (Davis, 1972; Akins and Dubey, 2008; Hong et al., 2010). In general, indirect labeling can be divided into two major classes, gene modification and external label. Gene modification is suitable for long-term tracking because the labeled genes can be steadily expressed in the next generation (Chan et al., 2017; Su et al., 2019). However, the imaging technique of gene modification is single and only being detected by a tracking fluorophore leading to the limitation of tissue penetration (Herschman, 2004; Loewen et al., 2004). In addition, the labeled genes are derived from exosomes, leading to immune incompatibility (Zheng et al., 2017). Another indirect labeling method, external label, can overcome the obstacle of the foreign gene by introducing additional contrast agents, but it still has some limitations (Crabbe et al., 2010; Li et al., 2013; Hachani et al., 2017). Because the introduction of contrast agents brings some new problems, including the reduction effect of photo-signal over time, optical interference is induced by tissue autofluorescence, incompatibility, cytotoxicity, and low labeling efficiency (Bellin, 2006; Cosgrove, 2006; Lusic and Grinstaff, 2013). Thus, an appropriate contrast agent should be developed and utilized to break through these limitations.

Nanoparticles (NPs), the diameter of which ranges from 1 to 100 nm, cover a diverse range of chemical composition with an equally diverse number of applications (Khan et al., 2019). Due to their nanoscale dimensions, NPs can easily transport across cell membrane and reach the crucial organelles including the endoplasmic reticulum, mitochondria, and nucleus (McNamara and Tofail, 2017). Moreover, a high surface area-over-volume ratio augments their interaction with cellular components (Bhirde et al., 2011; Edmundson et al., 2013). Furthermore, the morphology and size of NPs determine their physicochemical properties leading to the differences in cellular uptake and interaction with biological tissues, which could not be achieved by bulk materials (Barua and Mitragotri, 2014). As a result, NPs have been regarded as a promising contrast agent. Nowadays, nanotechnology in medical science has been regarded as a great breakthrough in this century (Zhang et al., 2008; Lin, 2015). Importantly, some special NPs have unique magnetic and/or optical properties, which can offer real-time imaging *in vivo* for tracking the transplanted stem cells, showing a strong potential in breaking through the obstacle of external label (Rhyner et al., 2006; Hahn et al., 2011). These NPs for tracking stem cells include organic, inorganic, and composite NPs, and the representative examples have magnetic NPs and photoacoustic NPs (Van Den Bos et al., 2003; Ferreira, 2009; Wang et al., 2012b; Gao et al., 2013; Accomasso et al., 2016; Riera et al., 2019). The transplanted stem cells labeled by these NPs can be detected by multiple imaging methods, such as magnetic resonance imaging (MRI), nuclear imaging [including single-photon emission computed tomography imaging (SPECT) and positron emission tomography-computed tomography (PET-CT)], and photoacoustic imaging (Sutton et al., 2008; Andreas et al., 2012; Cheng et al., 2016; Guo et al., 2019b; Patrick et al., 2020). However, there are still many problems to be addressed in the actual application process for NPs. First, NPs for tracking stem cells *in vivo* always require good cellular uptake and efficient internalization. Therefore, the surface chemistry of NPs should be importantly considered (Accomasso et al., 2016). In most cases, NPs with positive charge have the improvement of cellular uptake due to electrostatic interaction, leading to an accelerated internalization (Saha et al., 2013; Behzadi et al., 2017). Certainly, NPs with positive charge also may delay their internalization process, due to the influence on the adsorption of some specific proteins (Jambhrunkar et al., 2014). Second, importantly, before use, NPs should be comprehensively characterized for their composition and purity under different conditions, in order to evaluate their toxic effects on cells and ensure safety *in vivo* (Chandran et al., 2017). The reasons are that their toxic effects on cells may highly depend on the unique characteristics of NPs themselves. What is more, compared with bulk material, NPs have higher surface area-to-volume ratio and surface reactivity, which are more susceptible to the environment, so they should be modestly employed (Alkilany and Murphy, 2010; Lankoff et al., 2012). In addition, close attention should be paid to the agglomeration of NPs, which may lead to bad cellular uptake and further induced cytotoxicity (Gliga et al., 2014).

The internalization of NPs is influenced by their charge, size, shape, and the ability of cellular uptake, due to the force of their internalization driven by endocytosis. After internalization by endocytosis, NPs will pass environments of lysosomes and peroxisomes where the acidic material and oxidative substance could entirely degrade them. Possibly, a part of them may undergo exocytosis or escape to other intracellular locations (Chen et al., 2013; Calero et al., 2014).

## Nanoparticles for Tracking MSCs and the Potential Treatment of Cardiovascular Diseases

Cardiovascular diseases (CVDs), with high incident, are one of the major causes of human deaths worldwide and responsible for more than 17.7 million deaths in 2015, according to the World Health Organization (Zampelas and Magriplis, 2020). Therefore, developing an effective means for treating CVDs is of paramount urgency (Behlke et al., 2020). Currently, the means for treating CVDs include, but are not limited to, surgical treatment, delivering the targeted drug, and the repair and regeneration of the cardiac tissues (Godin et al., 2010; Cui et al., 2016; Deng et al., 2020). However, surgical treatment and targeted drug delivery only can delay and relieve the progression of CVDs but are unable to solve the inherent problems due to the deficiency of the regeneration for dead heart cells. In regenerative medicine, adult stem cell-based therapies have been shown as a promising potential for treating cardiovascular diseases in preclinical trials because the repair and regeneration of the cardiac tissues can be expected to solve the differentiation of stem cells into heart cells, thereby, offering improved efficacy (Kastrup, 2011). In these cells, MSCs play an important role in the therapy of CVDs, due to their ability of self-renewal and differentiating into various types of cells, including cardiovascular cells (Caplan, 2009; Ye et al., 2014). Moreover, after transplantation of MSCs, they can generate a structure resembling cardiac myocytes and exert anti-inflammatory effects, which can protect the myocardium, further improving the repair of the cardiovascular system. Notably, MSCs have been demonstrated by preclinical studies to have a strong ability to generate blood vessels with minimal adverse effects on the tissues near the injected region. Therefore, the transplantation of MSCs is potentially promising to improve the treatment of CVDs (Trivedi et al., 2010; Goradel et al., 2018; Guo et al., 2020). Despite its great potential for CVD treatment, MSC-based therapies still face some challenges, for example, the real-time tracking for MSC fate after their transplantation, which can provide information such as cell differentiation, the role that MSCs play in vascular repair, and mechanisms of cardiovascular regeneration to guide therapy process and improve therapeutic effect.

Cell imaging is an ideal method for real-time tracking of MSCs, but requires a suitable contrast agent to label MSCs. NPs are a group of contrast agent, which have been widely applied in MSC tracking (Zhang and Wu, 2007; Fu et al., 2011; Liang et al., 2013; Santoso and Yang, 2016; Yi et al., 2017; Guo et al., 2019a). Especially, MSCs labeled by magnetic and/or optical NPs can directly be imaged to offer a real-time tracking *in vivo* (Jing et al., 2008; Betzer et al., 2014; Wu et al., 2014; Perez et al., 2017). These NPs include superparamagnetic iron oxide (SPIO) NPs, gadolinium (Gd)-based NPs, gold (Au)-based NPs, quantum dots (QDs)-based NPs, upconversion (UC)-based NPs, silicon-based NPs, and several other classes of NPs. Here, we will summarize and discuss in detail NPs for labeling and tracking MSCs *in vivo*, shown in Table 1.

**TABLE 1:** Comparison between nanoparticles for tracking mesenchymal stem cells (MSCs).

**Example**	**Types of nanoparticles (NPs)**	**Preparation**	**Imaging modality**	**Types of MSCs**	**Advantages**	**Disadvantages**	**Main results**
Lu et al., 2007; Andreas et al., 2012	Superparamagnetic iron oxide (SPIO) NPs	Easy	T2-weighted magnetic resonance imaging (MRI)	Human MSCs	Obvious contrast	Low cell-labeling efficiency for MSCs; necessary to functionalize SPIO NPs	No apparent influences on viability of MSCs
Babic et al., 2008; Kim et al., 2011; Szpak et al., 2013; Lewandowska-Łańcucka et al., 2014; Park et al., 2014; Liao et al., 2016; Rawat et al., 2019; Gu et al., 2018; Lee et al., 2020	SPIO NPs	Complex	T2-weighted MRI	Human MSCs; mouse MSCs	Obvious contrast; enhancement of cellular internalization	Induce precipitation of NPs; perturb the cell membrane	No apparent influences on viability of MSCs
Zhang et al., 2010	SPIO NPs	Easy	Proton (^1^H) MRI images	Human MSCs	High sensitivity of cell detection	Difficult to accurately quantify cell population	No apparent influences on viability of MSCs
Sehl et al., 2019	SPIO NPs	Complex	Proton (^1^H) MRI images	Mouse MSCs	High sensitivity of cell detection; quantify the persistence of transplanted MSCs		No apparent influences on viability of MSCs
Sherry et al., 1988; Ratzinger et al., 2010	Gd-based NPs	Easy	T1-weighted	Human MSCs	Distinguish some similar low signal	Low cellular uptake for MSCs	No apparent influences on viability of MSCs
Kim et al., 2015b; Santelli et al., 2018	Gd-based NPs	Easy	T1-weighted MRI	Human MSCs	Distinguish some similar low signal; long-term tracking		No apparent influences on viability of MSCs
Huang et al., 2020	Au-based NPs	Easy	Photoacoustic imaging and CT imaging	Mouse MSCs	Detected in deep tissue at a high resolution; directly labeled; excellent biocompatibility		No apparent influences on viability of MSCs
Chen G. et al., 2015; Li et al., 2016	QD-based NPs	Complex	Fluorescence imaging	Human MSCs	Longer lifetime than traditional fluorescence dye; photochemical stability	Unsatisfied cytotoxicity and stochastic blinking; the contradiction of sensitivity and definition	No apparent influences on viability of MSCs
Idris et al., 2009; Ang et al., 2011; Zhao et al., 2013; Chen X. et al., 2015; Ma et al., 2016	UC-based NPs	Complex	UC luminescence imaging	Human MSCs	Detected in deeper tissue; more stable and higher definition	Poor biocompatibility; low uptake for cellular	No apparent influences on viability of MSCs
Huang et al., 2005; Chen and Jokerst, 2020; Yao et al., 2020	Silicon-based NPs	Complex	Fluorescence imaging	Human MSCs	Excellent biocompatibility and chemical inertness; easily modified by bioconjugation; good cellular uptake		No apparent influences on viability of MSCs
Chen et al., 2019	Silicon-based NPs	Complex	Photoacoustic imaging	Human MSCs	Good biocompatible and cells tracking capacity; long time		No apparent influences on viability of MSCs
Gao et al., 2016	Other-based NPs	Complex	Aggregation-induced emission imaging	Mouse bone marrow-derived MSCs	Possessing long-term tracking and strong anti-photobleaching ability		No apparent influences on viability of MSCs
Yin et al., 2018	Other-based NPs	Complex	Photoacoustic imaging	Human MSCs	High signal-to-noise; deeper tissue imaging		No apparent influences on viability of MSCs

### Superparamagnetic Iron Oxide Nanoparticles

Generally, SPIO NPs are a kind of NPs with a core–shell structure, in which the core is composed of an iron oxide and then is covered by a coating layer as the shell. The coatings layer alters the surface properties of SPIO NPs, and the iron oxide core provides its magnetic properties (Singh et al., 2010; Mahmoudi et al., 2011). Due to unique magnetic properties, SPIO NPs have the ability to generate a strong inhomogeneous field leading to the contraction of T2 relaxation time, thereby, generating obvious contrast in T2-weighted MRI, and are generally used as a T2-contrast agent (Andreas et al., 2012; Li et al., 2013; Bull et al., 2014; Santoso and Yang, 2016; Jiang et al., 2019). So far, SPIOs are the only commercial NPs, several formulations of which have already been approved by the Food and Drug Administration and have been regulated for clinical applications (Wang et al., 2013). MSCs labeled by SPIO NPs can be tracked by MRI, providing a non-invasive method to monitor the fate of transplanted MSCs *in vivo* (Li et al., 2013; Bull et al., 2014; Guldris et al., 2017; Xie et al., 2019; Elkhenany et al., 2020).

However, MSCs lack phagocytic capacity, and the surface charge of SPIO NPs are negative, which is a similar charge to that of the cellular membrane. Therefore, the internalization of SPIO NPs is limited, leading to a low efficiency of cell labeling. In order to solve these problems, it is necessary to functionalize SPIO NPs by introducing positive charges or other cell membrane-penetrating moieties on the surface (Lu et al., 2007; Andreas et al., 2012). Moreover, these unique functionalized surfaces also increase the stability of SPIO NPs in hydrophilic conditions, as well as are used as a coating layer for delaying the degradation of the iron oxide core (Barrow et al., 2015). For example, the uses of cationic polymers such as poly-L-lysine (PLL) and chitosan have commonly been utilized to improve the cellular internalization of SPIO NPs and protect them from being easily degraded *in vivo* (Babic et al., 2008; Szpak et al., 2013; Lewandowska-Łańcucka et al., 2014; Park et al., 2014). As an MRI probe for tracking MSCs, PLL-SPIONs were more efficient and safer than naked iron oxide NPs, which was in accordance with the fact that their *R*_2_ value was higher than that of uncoated nanoparticles. However, the highly positive charge of PLL induced the precipitation of NPs and perturbed the cell membrane. Liu et al. (2011) used polyethylenimine with a low molecular weight (2 kDa) to wrap SPIO NPs and obtain PEI–SPIO NPs, which could be readily internalized by MSCs for long-term tracking (19 days). Compared with single SPIO NPs, the modified SPIO NPs hold a controlled clustering structure, leading to much higher T2 relaxivity. Moreover, Lewin et al. (2000) used a short HIV-Tat peptide to functionalize SPIO NPs, which could be effectively engulfed by hematopoietic and neural progenitor cells. The results suggested that the cellular uptake of iron can reach an extremely high level (10–30 pg per cell) showing effective cell labeling and MRI tracking. However, NPs functionalized by cationic polymer or molecules may cause a certain level of toxicity, although it would drastically enhance cellular uptake of NPs. Therefore, some researchers (Kim et al., 2011; Liao et al., 2016) utilized 2-aminoethyl-trimethyl ammonium (TMA) to modified SPIO NPs. It was found that TMA–SPIO NPs could easily and efficiently label MSCs to monitor their fate *in vivo*, and the labeled MSCs showed low cytotoxicity. In another work, Gu et al. (2018) synthesized PA–SPIO NPs using a self-assembled lipopeptide amphiphile (PA) to modify the surfaces of SPIO NPs for labeling mouse MSCs. The modified NPs showed the enhancement of labeling efficiencies and the improvement of their contrast by shortening the relaxation time. In addition, these NPs exhibited excellent dispersibility and stability in water. More importantly, MSCs labeled by the PA–SPIO NPs were transplanted into mouse, showing no adverse effects on the osteogenic and adipogenic differentiation. Rawat et al. (2019) synthesized SPIO NPs and then used them to label MSCs. The labeled MSCs could be tracked by MRI to understand their fate *in vivo*. Moreover, these labeled MSCs still maintained differentiation potential. Lee et al. (2020) used poly lactic-co-glycolic acid (PLGA) to modify SPIO NPs, and then utilized fluorescent dye Cy5.5 to functionalize the prepared NPs for labeling and tracking MSCs to investigate the interactions between PLGA–SPIO NPs and MSCs. The results showed that the prepared NPs had no apparent influences on the survival and differentiation of MSCs when the concentration was at 40 μg/ml for more than 96 h, demonstrating that the internalization pathway of MSCs is via endocytosis.

**FIGURE 1:**
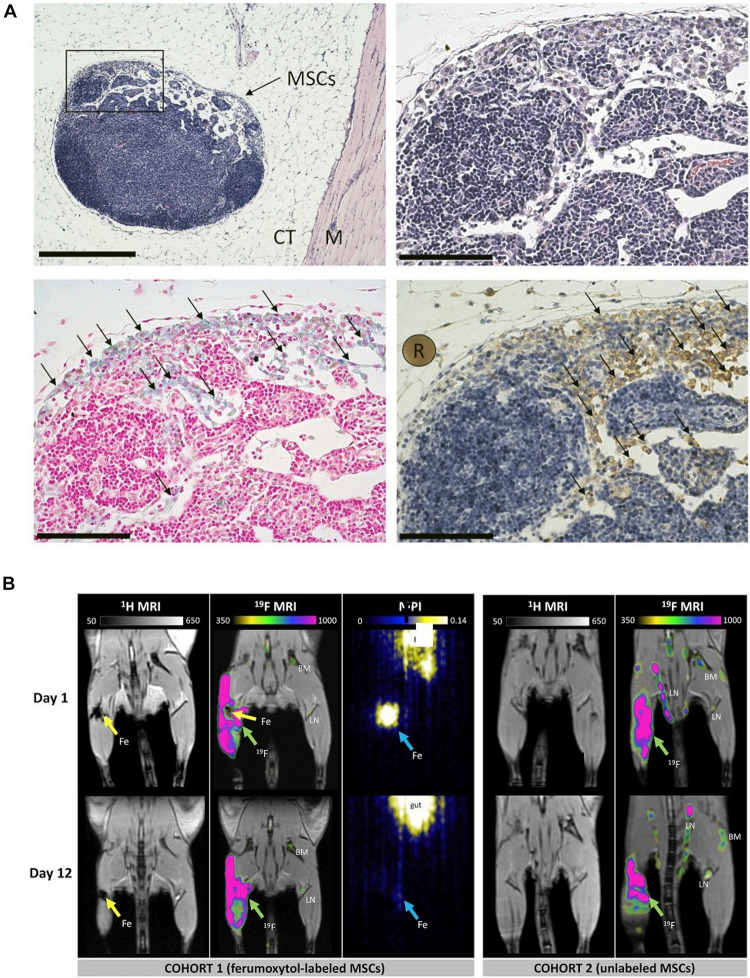
**(A)** Histological validation showing the presence of MSCs surrounded in connective tissue (CT) and muscle (M) in hematoxylin and eosin at × 10 magnification (scale bar 500 μm). **(B)** *In vivo* proton (^1^H)/fluorine 19 (^19^F) magnetic resonance imaging (MRI) and magnetic particle imaging (MPI) (adapted Sehl et al., 2019).

In proton (^1^H) MRI images, SPIO NP-labeled cells appear as signal void regions (Miguel et al., 2007). This effect gives rise to enhance the sensitivity of cell detection, but poses challenges for accurate quantification of the cell population as well. Another limitation for SPIO NP-based cell tracking is the lack of specificity in some tissues. It is ambiguous to identify these cells *in vivo*, as other regions in anatomic MRI appear as dark as well (Zhang et al., 2010). To overcome these limitations, Sehl et al. (2019) utilized the combination of iron-based MRI, ^19^F MRI, and MPI cellular imaging technologies to monitor and quantify the persistence of the transplanted MSCs and infiltrate macrophages *in vivo*, shown in Figure 1. First, MSCs were labeled with iron oxide NPs (ferumoxytol) and then implanted into the hind limb muscle of 10 C578/6 mice. Finally, a perfluorocarbon agent was administered intravenously for uptake by phagocytic macrophages *in situ*. The ferumoxytol-labeled MSCs were detected by proton (H^1^) MRI and magnetic particle imaging (MPI). Perfluorocarbon-labeled macrophages were detected by fluorine-19 (19F) MRI. These three modalities are complementary and provide integrated information (specificity, sensitivity, and quantification of cell number). They proposed that these cellular imaging techniques could be used to monitor MSC engraftment over time and detect the infiltration of macrophages at transplant sites.

### Gadolinium-Based Nanoparticles

T1-weighted sequences are often collected before and after infusion of T1-shortening MR contrast agents, so as to reduce the T1 relaxation times of ^1^H atoms in water molecules and produce hyperintensities in T1-weighted images appearing white (Na and Hyeon, 2009). Currently, Gd-based NPs are the most widely used T1-contrast agent for labeling and tracking stem cells (Ni and Chen, 2015). Compared with T2-contrast agent of SPIO NPs, T1-contrast agent of Gd-based NPs can distinguish some similar low signal resulting from intrinsic signal in tissue or hemorrhage, due to their capability of generating a bright positive signal. Therefore, a T1-contrast agent generally is predominately employed as a probe in detecting the transplanted stem cells in a low-signal region (Na and Hyeon, 2009; Zhu et al., 2013; Robert et al., 2015).

**FIGURE 2:**
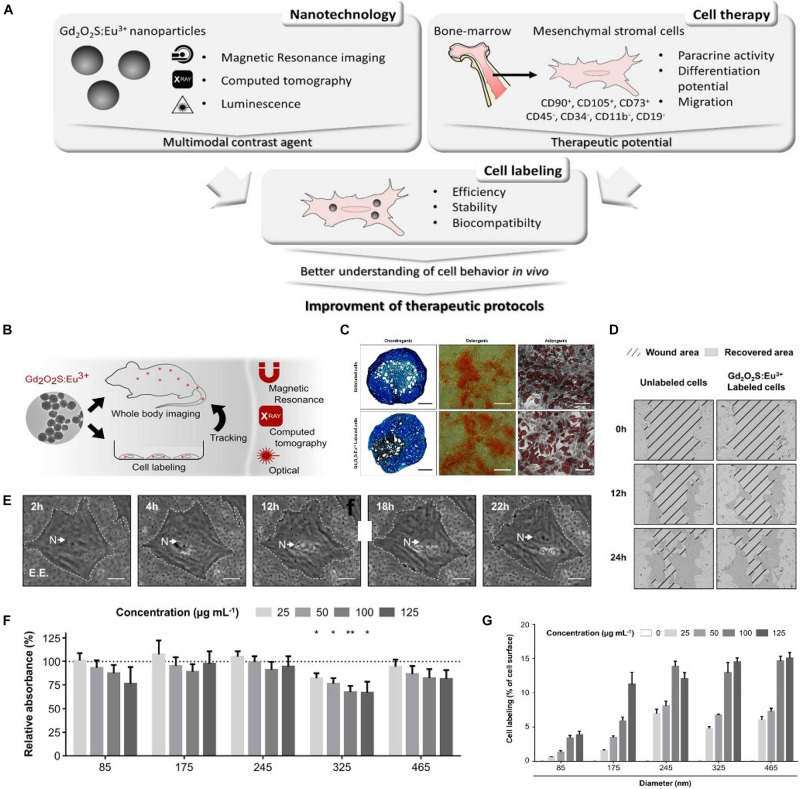
**(A)** Schematic representation of nanoparticle potential in cell therapy and future impact. **(B)** Multimodality imaging for tracking mesenchymal stem cells (MSCs). **(C)** Differentiation of MSCs (scale bar 150 μm). **(D)** Representative phase-contrast images of wound closing. **(E)** Time-lapse of labeling extracted from video microscopy acquisition. **(F)** Viability of MSCs after 24-h Gd_2_O_2_S:Eu^3+^ labeling evaluated by MTT. **(G)** Quantification of MSC labeling with Gd_2_O_2_S:Eu^3+^ expressed as the percentage of cell surface area occupied by NPs (adapted by Santelli et al., 2018).

Gd-based NPs usually are complex NPs, being composed of Gd^3+^ and their chelating ligand of which diethylenetriamine pentaacetic acid (DTPA) is the most common (Sherry et al., 1988; Ratzinger et al., 2010). Modified by DTPA, GD-based NPs reduced cytotoxicity due to the enhancement of hydrophilicity, but also weakened the interaction with the cell membrane leading to low cellular uptake for MSCs. Furthermore, their labeling efficiency was low resulting from the poor targeting ability of MSCs. Considering these problems, Tseng et al. (2010) prepared Gd-based NPs used as an MRI contrast agent to label and track human MSCs. Due to the modification of hexanedione, the biocompatibility of the prepared Gd-based NPs was improved. Moreover, compared with Gd-based NPs modified by DPTA, the prepared Gd-based NPs showed more excellent cellular uptake for MSCs and could be easily detected *in vivo* due to the shortening of T1 relaxation times. In another work, Kim et al. (2015b) reported a Gd-based NP to label MSCs, in which Gd^3+^ was chelated with a pullulan derivative ligand. It was found that the prepared Gd-based NPs could dramatically enhance cellular uptake for MSCs, leading to the enhancement of internalization efficiency from 32 ± 2 to 98 ± 4 pg Gd/cell. Moreover, the labeled MSCs achieved a long-term tracking of 21 days *in vivo*, due low signal attenuation. Cai et al. (2017) fabricated Gd-based NPs of 7 nm for tracking MSCs by utilizing melanin to modify Gd^3+^. The results showed that Gd-based NPs modified by melanin increased the stability of MRI contrast agent and shorten T1 relaxation time, compared with traditional Gd-based NPs modified by DTPA. The labeled MSCs achieved long-term tracking for more than 4 weeks, due to the enhancement of labeling efficiency. Importantly, the labeled MSCs had no apparent cytotoxicity at a proper concentration of 800 μg/ml. However, preclinical studies suggested that for tracking MSCs *in vitro* and *in vivo*, the single-imaging technique was difficult to complete, and the integration of multiple labeling means often was required. Consequently, Santelli et al. (2018) developed multimodality imaging to track MSCs labeled by Gd-based NPs, shown in Figure 2. The GD-based NPs composed of spherical europium-doped gadolinium oxysulfide (Gd_2_O_2_S:Eu^3+^) could be detected by MRI, X-ray imaging, and photoluminescence imaging. In the *in vitro* test, the number of MSCs labeled by Gd_2_O_2_S:Eu^3+^ was up to 2,500, showing feasible cell tracking. Moreover, the results suggested that the effects of the NPs on viability, proliferation, migration, and differentiation of the transplanted MSCs were innocuousness.

### Gold-Based Nanoparticles

Due to their cytocompatibility and strong optical absorption in the near-infrared region, Au-based NPs are potential contrast agents of photoacoustic imaging. In addition, Au-based NPs can be detected in deep tissue at 2 cm at a high resolution of 100 μm, so they are emerging as an alternative method for tracking cells *in vivo* (Murphy et al., 2008; Giljohann et al., 2010). More importantly, MSCs can be directly labeled by Au-based NPs, so their differentiation after transplantation *in vivo* can be detected using photoacoustic imaging *in vivo* (Turjeman et al., 2015). Donnelly et al. (2018) utilized Au-based NPs as a photoacoustic contrast agent for real-time tracking MSCs to guide their delivery. It was found that the labeled MSCs could be detected by ultrasound/photoacoustic imaging in the spinal cord. In another work, Dhada et al. (2019) prepared Au-based NPs to label and track MSCs by photoacoustic imaging, which was composed of gold nanorods and IR775. The results suggested that cell death also could be detected because IR775 was sensitive to reactive oxygen species (ROS). Therefore, the viability of the transplanted MSCs *in vivo* was quantificationally measured. Moreover, Laffey et al. (2020) studied the viability of MSCs labeled by Au-based NPs in a frozen environment. The results showed that the labeled MSCs undergoing the process of freeing, storing, and thawing for 2 months still could be detected by photoacoustic imaging, and their differential ability also had no apparent difference with the unlabeled MSCs.

**FIGURE 3:**
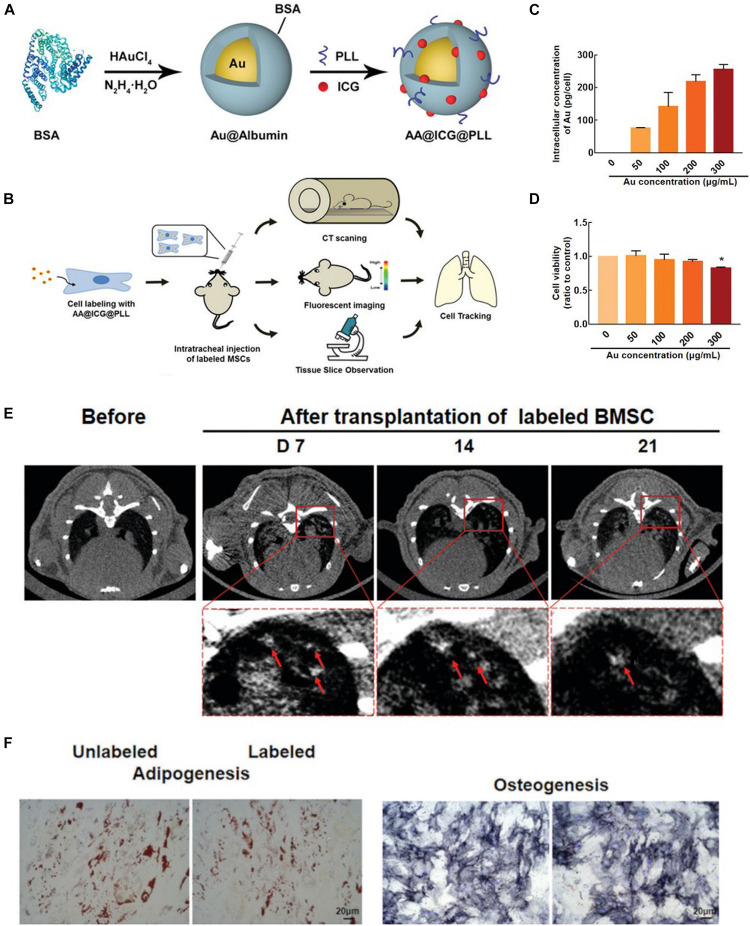
**(A)** Schematic illustration of the synthesis of AA@ICG@PLL gold (Au)-based nanoparticles (NPs). **(B)** Experimental design for tracking AA@ICG@PLL-labeled MSCs in a silica-induced PF mouse model. **(C)** Intracellular Au content measured by ICP-MS. **(D)** Relative viability of BMSCs labeled with AA@ICG@PLL NPs at various Au concentrations. **(E)** *In vivo* computed tomography (CT) images of AA@ICG@PLL-labeled BMSCs at 7, 14, and 21 days after transplantation. **(F)** Bright-field images of Oil Red O staining and ALP staining of NP-loaded and unloaded BMSCs. Scale bar = 20 mm (adapted by Huang et al., 2020). ^∗^*p* < 0.05 compared with the unlabeled group.

Certainly, Au-based NPs also can be detected by computed tomography (CT), due to the high X-ray absorption. Thus, they are used as a CT maker to label and track MSCs. For example, Betzer et al. (2017) used Au-based NPs as labeling agents to longitudinally and quantitatively track MSCs *in vivo*. It was found that the labeled MSCs were detected for up to 24 h by CT, showing a long-term tracking. Nafiujjaman and Kim (2020) reported a biocompatible Au-based NPs used as a CT maker, in which Au NPs were modified by poly-L-lysine (PLL) to change the charge properties of surface enhancing the cellular uptake. The MSCs labeled by Au-based NPs could be detected by CT and showed high labeling efficiency. Huang et al. (2020) synthesized Au-based NPs (AA@ICG@PLL) with dual-modal imaging (CT and near-infrared fluorescence) to label and track MSCs of mice *in vivo*, shown in Figure 3. Due to modification by indocyanine green (ICG) and poly-L-lysine (PLL), AA@ICG@PLL showed excellent cellular uptake for MSCs and biocompatibility. It was found that the labeled MSCs could be tracked via dual-modal imaging for more than 21 days, and importantly, the prepared NPs had anti-inflammatory properties.

### Quantum Dot-Based Nanoparticles

QD-based NPs are semiconductor NPs with II–VI or III–V elements, which can emit particular frequency light after being stimulated (Jaiswal et al., 2004). The emitted light frequency depends on the size and shape of QD-based NPs. Therefore, QD-based NPs show different colors (Seleverstov et al., 2006). In general, the fluorescence lifetime of QD-based NPs is longer than traditional fluorescence dye (Bailey et al., 2004). In addition, compared with fluorescent dye, QD-based NPs are easier to be detected due to the non-overlapping of absorption and emission (Mazumder et al., 2009). Specifically, QD-based NPs have broad absorption of continuous distribution, but show narrow and symmetrical properties (Yukawa and Baba, 2017). More importantly, they have the advantage of photochemical stability. As a result, QD-based NPs have been used as contrast agents for long-term tracking of MSCs *in vivo*. However, QD-based NPs also have some disadvantages, such as unsatisfied cytotoxicity and stochastic blinking. Moreover, the augmentation of QD-based NPs in dosage increases the sensitivity of cell imaging, but may decrease their definition (Shah et al., 2007; Liang et al., 2013).

**FIGURE 4:**
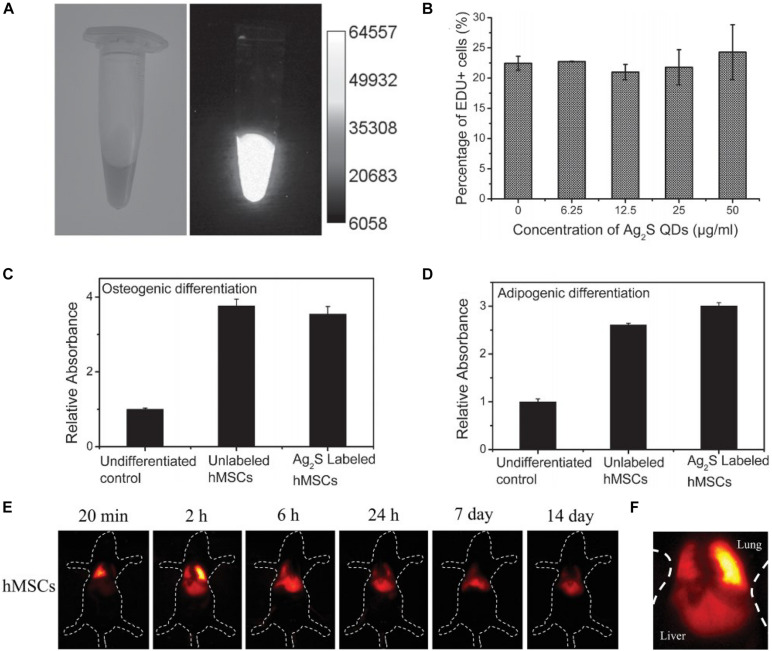
**(A)** A photoluminescence image (PA) of the Ag_2_S QD-labeled human MSC solution at a density of 4 × 10^6^ cell ml^–1^. **(B)** Cell proliferation. Quantification of **(C)** osteogenic and **(D)** adipogenic differentiation by measuring the absorbance of Oil-Red O and alizarin red extracted from cell lysates at 404 nm, respectively. **(E)** The time course of the *in vivo* near-infrared (NIR) PL images of a healthy mouse after transplantation of Ag_2_S QD-labeled human MSCs. **(F)** Higher magnification NIR PL image of mice transplanted with human MSCs only after 2 h (adapted from Chen G. et al., 2015).

Hsieh et al. (2006) reported a QD-based NP to label human MSCs, in which CdSe was used as the core, and the shell was covered by ZnS. In addition, the commercial QD-based NPs also were developed and utilized as contrast agents to label and track the cell distributions. However, these QD-based NPs showed unsatisfied biocompatibility and cytotoxicity, so they had to be modified before use. In recent years, Li et al. (2016) prepared QDs-based NPs (RGD-β-CD-QDs) to label and track human MSCs, which were composed of QDs, β-cyclodextrin (β-CD), and Cys-Lys-Lys-ArgGly-Asp (CKKRGD) peptide. The QDs modified by β-CD enhanced the cellular uptake and facilitated the differentiation of MSCs, due to the small molecule dexamethasone and siRNA carried by β-CD. More importantly, the labeled MSCs could be detected for up to 3 weeks. Chen G. et al. (2015) reported a AgS_2_ QD-based NP for tracking human MSCs *in vivo* by utilizing fluorescence imaging, shown in Figure 4. It was found that the prepared NPs with good biocompatibility could be detected in the second near infrared, and the stability of fluorescence reached up to 30 days. Furthermore, the labeled MSCs was dynamically visualized at high resolution of 100 ms for more than 14 days. Chetty et al. (2019) prepared CuInS_2_-ZnS QD-based NPs to label umbilical cord-derived MSCs, and the labeling efficiency reached 98% due to the prepared QD-based NPs displaying high photoluminescence quantum yield of 88%. In addition, the labeled MSCs showed no apparent influences on stemness and had the ability of long-term tracking.

### Upconversion-Based Nanoparticles

UC-based NPs, usually being lanthanide-doped nanocrystals, can undergo a photon UC process in which the sequential absorption of two or more photons leads to anti-Stokes type emission of a single higher-energy photon (Wang et al., 2010). Due to the unique properties of being sensitive to the near-infrared light, UC-based NPs can be detected in deeper tissue compared with traditional contrast agents with fluorescence. More importantly, the imaging of UC-based NPs is more stable and has higher definition (Ang et al., 2011; Chen X. et al., 2015).

Idris et al. (2009) reported a contrast agent of UC-based NPs to label and track MSCs, which was composed of NaYF_4_:Yb, Er NPs, and silicon. The labeled MSCs could directly be imaged *in vivo* to dynamically visualize their behaviors, but the region imaged by confocal microscopy was small. Wang et al. (2012a) fabricated UC-based NPs to label MSCs, which was modified by polyethylene glycol (PEG) and oligo-arginine. MSCs labeled by the UC-based NPs were detected by fluorescence imaging *in vivo*, and the introduction of oligo-arginine improved the cellular uptake of the NPs, showing excellent labeling efficiency and high sensitivity of 10 cells. In another work, Cheng et al. (2013) developed a multifunctional UC-based NP, integrating magnetic properties into UC luminescence NPs. Utilizing the prepared NPs, the labeled MSCs can be tracked via fluorescence imaging and MRI, possessing highly effective sensitivity of 10 cells in mouse *in vivo*. Zhao et al. (2013) prepared a UC-based NP for labeling mouse MSCs, which was composed of polyethylenimine (PEI) and (α-NaYbF_4_:Tm^3+^)/CaF_2_, enhancing the detective depth *in vivo* due to absorption and emission for near-infrared light.

**FIGURE 5:**
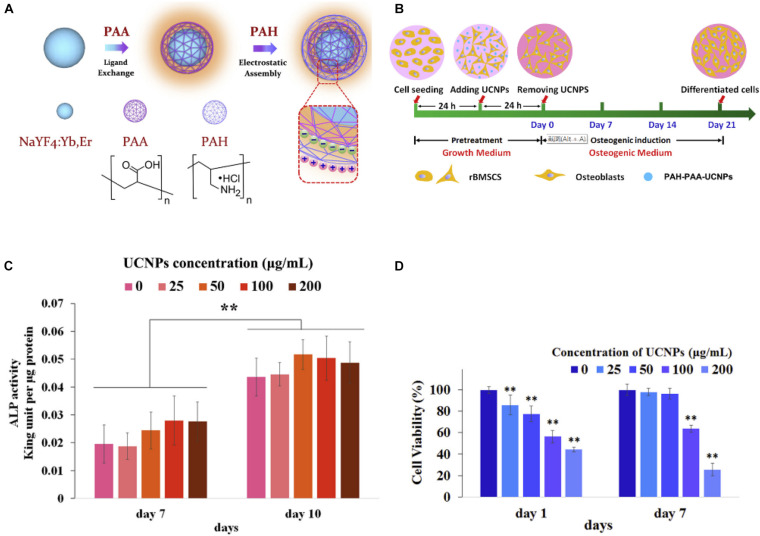
**(A)** Upconversion (UC)-based NPs synthesis. **(B)** Cell pretreatment and osteogenic differentiation process. **(C)** Normalized alkaline phosphatase activity expression by MSCs. **(D)** Cell viability (adapted by Ma et al., 2016). ^∗∗^*p* < 0.01 compared with control group.

In recent years, Ma et al. (2016) reported a UC-based NP, which was composed of NaYF_4_:Yb^3+^, Er^3+^ NPs, poly (acrylic acid) (PAA), and poly (allylamine hydrochloride) (PAH), as a fluorescence maker for tracking bone marrow MSCs *in vitro*, shown in Figure 5. The biocompatibility and cellular uptake of NaYF_4_:Yb^3+^, Er^3+^ NPs were highly enhanced, due to electrostatic interaction. Moreover, it was found that the effects of cellular uptake of UC-based NPs (≤50 μg/ml) on the osteogenic differentiation had no apparent difference by tracking the MSCs. Kang et al. (2018) prepared a UC-based NP with NIR-controllable properties to label MSCs. By a remote-controllable way, the stem cell differentiation was regulated. Furthermore, Ren et al. (2020) used ligand-free NaYF4:Yb/Er UC-based NPs to label and track mouse bone MSCs, demonstrating that the UC-based NPs at a proper concentration could enhance osteogenic differentiation.

### Silicon-Based Nanoparticles

Recently, silicon-based NPs have been demonstrated as a good contrast agent to label cells because they can be easily modified by bioconjugation (Shin et al., 2019). Furthermore, it has excellent biocompatibility and chemical inertness, thus, being able to serve as a probe for tracking cells *in vivo* (Huang et al., 2005; Hsiao et al., 2008). Silicon-based NPs, being simply divided into silica NPs and silicon carbide NPs, not only contain fluorescent properties but also are used as ultrasound agents (Chen and Jokerst, 2020; Yao et al., 2020).

**FIGURE 6:**
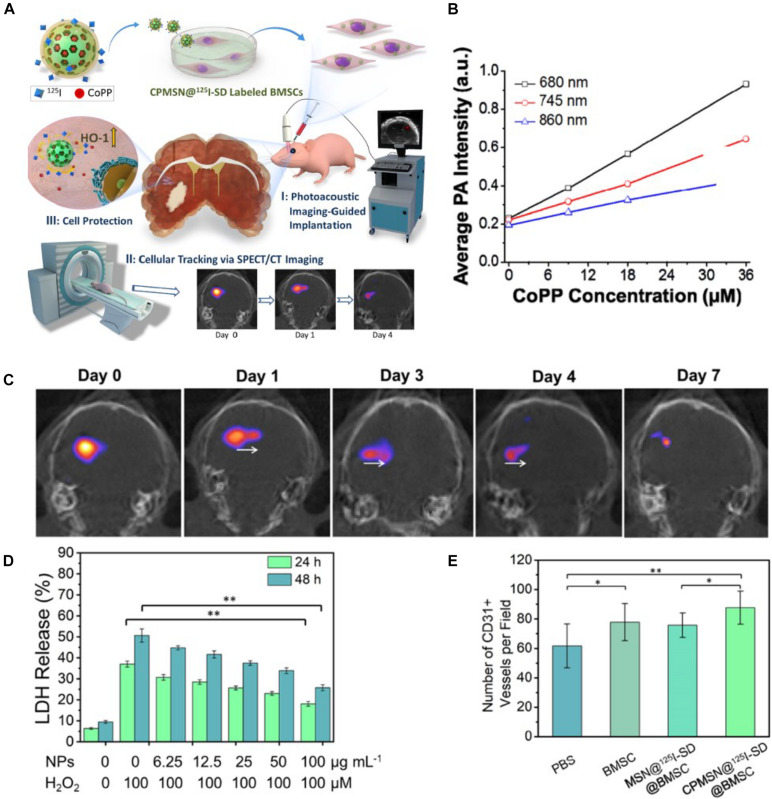
**(A)** Schematic illustrating the triple functionality of CPMSN@125I-SD for stem cell therapy of brain ischemia. **(B)** Corresponding average intensities of CPMSN@125I-SD with various cobalt protoporphyrin IX (CoPP) concentrations in the NIR region. **(C)** Single-photon emission computed tomography imaging (SPECT)/CT images of ischemic mouse brain tissue on different days (0–7 days) after intracerebral injection of the labeled BMSCs (500,000 cells). **(D)** Cell death assessment for MSCs after treatment with different concentrations of CPMSN@125I-SD (0–100 μg ml^–1^) and exposure to 100 μM H_2_O_2_ for 24 and 48 h. **(E)** Bar graph showing the quantification of the number of CD^31+^ cells (adapted from Yao et al., 2020). ^∗^*p* < 0.05; ^∗∗^*p* < 0.01.

Huang et al. (2005) prepared a mesoporous silica NP modified by fluorescein isothiocyanate, and the labeled MSCs could be detected by imaging to track their viability *in vivo*. Due to clathrin-mediated endocytosis, the NPs could be internalized into MSCs and showed good cellular uptake. In addition, the highly efficient labeling had no apparent influences on the viability of MSCs. In another work, Chen and Jokerst (2020) used silica NPs to label MSCs and then track the MSCs *in vivo* by ultrasound imaging. The results showed that silica NPs could significantly increase the ultrasound signal of MSCs *in vivo*. Yao et al. (2020) reported a unique core–shell NP in which the core is composed of cobalt protoporphyrin IX (CoPP)-loaded mesoporous silica NPs, and the shell is a ^125^I-conjugated/spermine-modified dextran polymer, to label and guide the transplantation of MSCs by PA imaging and SPCT nuclear imaging, shown in Figure 6. The obtained NPs not only could instantly image the transplantation of MSCs but also quantitatively tracked their migrations for a long time. Significantly, the NPs steadily released CoPP to increase the survival of MSCs in ischemic mice.

Another type of silicon-based NPs is silicon carbide NPs, which are commonly used as protecting layer due to their unique properties of being durable and chemically inert. Moreover, typically, silicon carbide NPs (≤10 nm) are utilized as contrast agents to label and track cells *in vivo* because of their photoluminescence resulting from quantum confinement effects. Chen et al. (2019) used three sizes of silicon carbide NPs to label MSCs, showing dual modality imaging (photoluminescence and photoacoustic imaging). When the size of the NPs is 620 nm, the labeled MSCs could be detected *in vitro* for more than 20 days. Moreover, the NPs also exhibited good biocompatibility and the capacity of cell tracking because the differentiated cells also could be imaged.

### Other Nanoparticles

**FIGURE 7:**
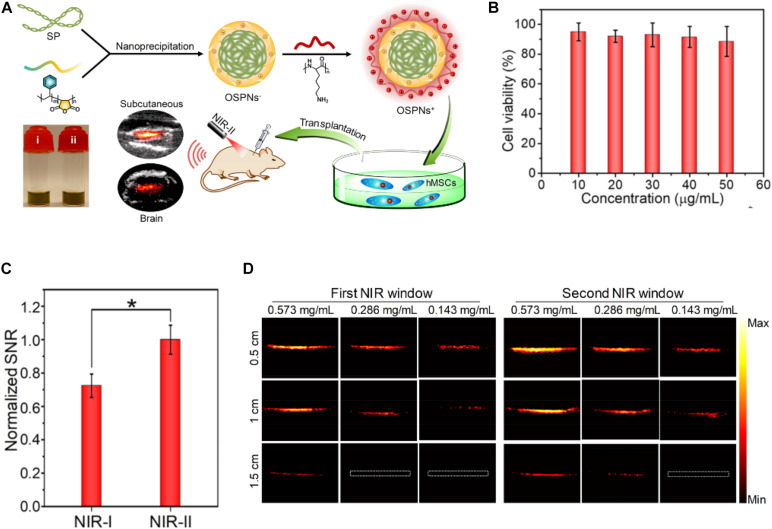
**(A)** Illustration of the preparation procedure of OSPNs^+^ and the photoacoustic labeling of human MSCs after transplantation. **(B)** The MTT assay of human MSCs treated by OSPNs^+^ for 12 h under various concentrations. **(C)** Normalized PA SNR of OSPNs^+^-labeled hMSCs implanted into mice brain under NIR-I (860 nm) or NIR-II (1,064 nm) light excitation (*p < 0.05). **(D)** PA imaging of aqueous OSPNs^+^ solutions (0.573, 0.286, and 0.143 mg/ml) (adapted from Yin et al., 2018).

Gao et al. (2016) prepared an aggregation-induced emission NP, the surface of which is modified by Tat peptide. The obtained NPs had highly efficient ability of labeling mouse bone marrow-derived MSCs, and the red emission could be detected for more than 12 passages, which were not achieved by traditional fluorescence, possessing long-term tracking and strong anti-photobleaching ability. More importantly, MSCs labeled by the NPs showed low cytotoxicity, and their viability and differentiation had no apparent influences. Lim et al. (2019) developed bicyclo nonyne (BCN)-conjugated glycol chitosan NPs (BCN-NPs) as a delivery system of dual-modal stem cell imaging probes. Yin et al. (2018) reported an organic semiconducting polymer NP (OSPN^+^) as a PA contrast agent for tracking MSCs, utilizing a second near-infrared (NIR-II) adsorption to broaden the limitation of conventional inorganic PA contrast agents and the narrow range of the wavelength in the first near-infrared (NIR-I) window, shown in Figure 7. The prepared cationic NPs showed the deeper tissue imaging due to the significantly higher signal-to-noise (SNR) and enhanced the cellular uptake for human MSCs because of their good biocompatibility, appropriate size, and optimized surface property.

## Problems and Prospects

Mesenchymal stem cell transplantation has been shown to be a strong potential for the treatment of CVDs. Many NPs have been fabricated and used as contrast agents by non-invasive imaging to track and monitor the transplanted MSCs, for providing more information to guide further therapy. However, one big issue associated with NPs for labeled MSCs is that NPs released from dead cells will be uptaken by macrophages, which give false-positive information *in vivo*. Maybe, we can label dead cells with other methods to exclude the false-positive information. Moreover, the single-imaging technique can be inefficient to meet all needs for tracking, and each of those has its own disadvantages. Although there are some multimodal imaging techniques, more multimodal labeling agent-based NPs should be developed in the future to optimize MSC dose and delivery route for the treatment of CVDs. Importantly, the poor targeted migration and low survival rate of MSCs transplanted into the cardiovascular should be solved.

## Author Contributions

HH completed the first draft of the manuscript. XD, ZH, and ZY offered the excellent advices and perfected the first draft. WH improved the first draft and finally finished the manuscript and provided the support of funds. All authors contributed to the article and approved the submitted version.

## Conflict of Interest

The authors declare that the research was conducted in the absence of any commercial or financial relationships that could be construed as a potential conflict of interest.

## References

[B1] Accomasso L. Gallina C. Turinetto V. Giachino C. J. (2016). Stem cell tracking with nanoparticles for regenerative medicine purposes: an overview. *Stem Cells Int.* 2016:7920358. 10.1155/2016/7920358 PMC4709786 26839568

[B2] Akins E. J. Dubey P. (2008). Noninvasive imaging of cell-mediated therapy for treatment of cancer. *J. Nucl. Med.* 49 180S–195S. 18523073 10.2967/jnumed.107.045971 PMC3690596

[B3] Alison M. Islam S. (2009). Attributes of adult stem cells. *J. Pathol.* 217 144–160. 10.1002/path.2498 19085991

[B4] Alkilany A. M. Murphy C. J. (2010). Toxicity and cellular uptake of gold nanoparticles: what we have learned so far? *J. Nanopart Res.* 12 2313–2333. 10.1007/s11051-010-9911-8 21170131 PMC2988217

[B5] Aly R. M. (2020). Current state of stem cell-based therapies: an overview. *Stem Cell Investig.* 7:8. 10.21037/sci-2020-001 32695801 PMC7367472

[B6] Andreas K. Georgieva R. Ladwig M. Mueller S. Notter M. Sittinger M. (2012). Highly efficient magnetic stem cell labeling with citrate-coated superparamagnetic iron oxide nanoparticles for MRI tracking. *Biomaterials* 33 4515–4525. 10.1016/j.biomaterials.2012.02.064 22445482

[B7] Andres R. H. Choi R. Steinberg G. K. Guzman R. (2008). Potential of adult neural stem cells in stroke therapy. *Regen. Med.* 3 893–905. 10.2217/17460751.3.6.893 18947311

[B8] Ang L. Y. Lim M. E. Ong L. C. Zhang Y. J. N. (2011). Applications of upconversion nanoparticles in imaging, detection and therapy. *Nanomedicine (Lond)* 6 1273–1288. 10.2217/nnm.11.108 21929461

[B9] Babic M. Horák D. Trchová M. Jendelová P. Glogarová K. Lesný P. (2008). Poly (L-lysine)-modified iron oxide nanoparticles for stem cell labeling. *Bioconjug Chem.* 19 740–750. 10.1021/bc700410z 18288791

[B10] Bailey R. E. Smith A. M. Nie S. (2004). Quantum dots in biology and medicine. *Physica E: Low-dimensional Systems Nanostruct.* 25 1–12.

[B11] Barrow M. Taylor A. Murray P. Rosseinsky M. J. Adams D. J. (2015). Design considerations for the synthesis of polymer coated iron oxide nanoparticles for stem cell labelling and tracking using MRI. *Chem. Soc. Rev.* 44 6733–6748. 10.1039/c5cs00331h 26169237

[B12] Barua S. Mitragotri S. (2014). Challenges associated with penetration of nanoparticles across cell and tissue barriers: a review of current status and future prospects. *Nano Today* 9 223–243. 10.1016/j.nantod.2014.04.008 25132862 PMC4129396

[B13] Behlke L. M. Lenze E. J. Carney R. M. (2020). The cardiovascular effects of newer antidepressants in older adults and those with or at high risk for cardiovascular diseases. *CNS Drugs* 34 1133–1147. 10.1007/s40263-020-00763-z 33064291 PMC7666056

[B14] Behzadi S. Serpooshan V. Tao W. Hamaly M. A. Alkawareek M. Y. Dreaden E. C. (2017). Cellular uptake of nanoparticles: journey inside the cell. *Chem. Soc. Rev.* 46 4218–4244. 10.1039/c6cs00636a 28585944 PMC5593313

[B15] Bellin M.-F. (2006). MR contrast agents, the old and the new. *Eur. J. Radiol.* 60 314–323. 10.1016/j.ejrad.2006.06.021 17005349

[B16] Betzer O. Meir R. Motiei M. Yadid G. Popovtzer R. (2017). “Gold nanoparticle-cell labeling methodology for tracking stem cells within the brain,” in *Proceedings of the Nanoscale Imaging, Sensing, and Actuation for Biomedical Applications XIV: International Society for Optics and Photonics*, (Bellingham, DC: SPIE).

[B17] Betzer O. Shwartz A. Motiei M. Kazimirsky G. Gispan I. Damti E. (2014). Nanoparticle-based CT imaging technique for longitudinal and quantitative stem cell tracking within the brain: application in neuropsychiatric disorders. *ACS Nano* 8 9274–9285. 10.1021/nn503131h 25133802

[B18] Bhirde A. Xie J. Swierczewska M. Chen X. J. N. (2011). Nanoparticles for cell labeling. *Nanoscale* 3 142–153. 20938522 10.1039/c0nr00493f PMC6454877

[B19] Bull E. Madani S. Y. Sheth R. Seifalian A. Green M. Seifalian A. M. (2014). Stem cell tracking using iron oxide nanoparticles. *Int. J. Nanomed.* 9:1641. 10.2147/ijn.s48979 24729700 PMC3976208

[B20] Bulte J. W. J. R. (2017). Science to practice: can MR imaging cell tracking of macrophage infiltration be used as a predictive imaging biomarker for transplanted stem cell rejection? *Radiology* 284 307–309. 10.1148/radiol.2017170536 28723288

[B21] Cahill K. S. Gaidosh G. Huard J. Silver X. Byrne B. J. Walter G. A. J. T. (2004). Noninvasive monitoring and tracking of muscle stem cell transplants. *Transplantation* 78 1626–1633. 10.1097/01.tp.0000145528.51525.8b 15591951

[B22] Cai W. W. Wang L. J. Li S. J. Zhang X. P. Li T. T. Wang Y. H. (2017). Effective tracking of bone mesenchymal stem cells in vivo by magnetic resonance imaging using melanin-based gadolinium3+ nanoparticles. *J. Biomed. Mater. Res. A* 105 131–137. 10.1002/jbm.a.35891 27588709

[B23] Calero M. Gutiérrez L. Salas G. Luengo Y. Lázaro A. Acedo P. (2014). Efficient and safe internalization of magnetic iron oxide nanoparticles: two fundamental requirements for biomedical applications. *Nanomedicine* 10 733–743. 10.1016/j.nano.2013.11.010 24333592

[B24] Caplan A. I. (2009). Why are MSCs therapeutic? new data: new insight. *J. Pathol.* 217 318–324. 10.1002/path.2469 19023885 PMC8793150

[B25] Caplan A. I. (2017). Mesenchymal stem cells: time to change the name! *Stem Cells Transl. Med.* 6 1445–1451. 10.1002/sctm.17-0051 28452204 PMC5689741

[B26] Carella A. M. Cavaliere M. Lerma E. Ferrara R. Tedeschi L. Romanelli A. (2000). Autografting followed by nonmyeloablative immunosuppressive chemotherapy and allogeneic peripheral-blood hematopoietic stem-cell transplantation as treatment of resistant Hodgkin’s disease and non-Hodgkin’s lymphoma. *J. Clin. Oncol.* 18 3918–3924. 10.1200/jco.2000.18.23.3918 11099321

[B27] Chan K. Y. Jang M. J. Yoo B. B. Greenbaum A. Ravi N. Wu W.-L. (2017). Engineered AAVs for efficient noninvasive gene delivery to the central and peripheral nervous systems. *Nat. Neurosci.* 20 1172–1179. 10.1038/nn.4593 28671695 PMC5529245

[B28] Chandran P. Riviere J. E. Monteiro-Riviere N. A. J. N. (2017). Surface chemistry of gold nanoparticles determines the biocorona composition impacting cellular uptake, toxicity and gene expression profiles in human endothelial cells. *Nanotoxicology* 11 507–519. 10.1080/17435390.2017.1314036 28420299

[B29] Chen F. Jokerst J. V. (2020). Stem cell tracking with nanoparticle-based ultrasound contrast agents. *Methods Mol. Biol.* 2126 141–153. 10.1007/978-1-0716-0364-2_13 32112386 PMC8045894

[B30] Chen F. Zhao E. R. Hu T. Shi Y. Sirbuly D. J. Jokerst J. V. (2019). Silicon carbide nanoparticles as a photoacoustic and photoluminescent dual-imaging contrast agent for long-term cell tracking. *Nanoscale Adv.* 1 3514–3520. 10.1039/c9na00237e 33313479 PMC7729840

[B31] Chen G. Tian F. Li C. Zhang Y. Weng Z. Zhang Y. (2015). In vivo real-time visualization of mesenchymal stem cells tropism for cutaneous regeneration using NIR-II fluorescence imaging. *Biomaterials* 53 265–273. 10.1016/j.biomaterials.2015.02.090 25890725

[B32] Chen X. Peng D. Ju Q. Wang F. J. (2015). Photon upconversion in core–shell nanoparticles. *Chem. Soc. Rev.* 44 1318–1330. 25058157 10.1039/c4cs00151f

[B33] Chen X. Tian F. Zhang X. Wang W. J. (2013). Internalization pathways of nanoparticles and their interaction with a vesicle. *Soft Matter.* 9 7592–7600. 10.1039/c3sm50931a

[B34] Cheng L. Wang C. Ma X. Wang Q. Cheng Y. Wang H. (2013). Multifunctional upconversion nanoparticles for dual-modal imaging-guided stem cell therapy under remote magnetic control. *Adv. Functional. Mater.* 23 272–280. 10.1002/adfm.201201733

[B35] Cheng S.-H. Yu D. Tsai H.-M. Morshed R. A. Kanojia D. Lo L.-W. (2016). Dynamic in vivo SPECT imaging of neural stem cells functionalized with radiolabeled nanoparticles for tracking of glioblastoma. *J. Nucl. Med.* 57 279–284. 10.2967/jnumed.115.163006 26564318 PMC5831675

[B36] Chetty S. S. Praneetha S. Govarthanan K. Verma R. S. Vadivel Murugan A. J. (2019). Noninvasive tracking and regenerative capabilities of transplanted human umbilical cord-derived mesenchymal stem cells labeled with I-III-IV semiconducting nanocrystals in liver-injured living mice. *ACS Appl. Mater. Interfaces* 11 8763–8778. 10.1021/acsami.8b19953 30741534

[B37] Cosgrove D. J. (2006). Ultrasound contrast agents: an overview. *Eur. J. Radiol.* 60 324–330. 10.1016/j.ejrad.2006.06.022 16938418

[B38] Crabbe A. Vandeputte C. Dresselaers T. Sacido A. A. Verdugo J. M. G. Eyckmans J. (2010). Effects of MRI contrast agents on the stem cell phenotype. *Cell Transplant* 19 919–936. 10.3727/096368910x494623 20350351

[B39] Cui Z. Yang B. Li R.-K. (2016). Application of biomaterials in cardiac repair and regeneration. *Engineering* 2 141–148. 10.1016/j.eng.2016.01.028

[B40] Davis W. C. (1972). H-2 antigen on cell membranes: an explanation for the alteration of distribution by indirect labeling techniques. *Science* 175 1006–1008. 10.1126/science.175.4025.1006 5009391

[B41] Deng Y. Zhang X. Shen H. He Q. Wu Z. Liao W. (2020). Application of the nano-drug delivery system in treatment of cardiovascular diseases. *Front. Bioeng. Biotechnol.* 7:489. 10.3389/fbioe.2019.00489 32083068 PMC7005934

[B42] Dhada K. S. Hernandez D. S. Suggs L. J. (2019). In vivo photoacoustic tracking of mesenchymal stem cell viability. *ACS Nano* 13 7791–7799. 10.1021/acsnano.9b01802 31250647 PMC7155740

[B43] Diekman B. O. Guilak F. J. (2013). Stem cell-based therapies for osteoarthritis: challenges and opportunities. *Curr. Opin. Rheumatol.* 25:119. 10.1097/bor.0b013e32835aa28d 23190869 PMC3616879

[B44] DiPersio J. F. Stadtmauer E. A. Nademanee A. Micallef I. N. Stiff P. J. Kaufman J. L. (2009). Plerixafor and G-CSF versus placebo and G-CSF to mobilize hematopoietic stem cells for autologous stem cell transplantation in patients with multiple myeloma. *Blood* 113 5720–5726. 10.1182/blood-2008-08-174946 19363221

[B45] Donnelly E. M. Kubelick K. P. Dumani D. S. Emelianov S. Y. (2018). Photoacoustic image-guided delivery of plasmonic-nanoparticle-labeled mesenchymal stem cells to the spinal cord. *Nano Lett.* 18 6625–6632. 10.1021/acs.nanolett.8b03305 30160124

[B46] Edmundson M. Thanh N. T. Song B. J. T. (2013). Nanoparticles based stem cell tracking in regenerative medicine. *Theranostics* 3 573–582. 10.7150/thno.5477 23946823 PMC3741606

[B47] Elkhenany H. Abd Elkodous M. Ghoneim N. I. Ahmed T. A. Ahmed S. M. Mohamed I. K. (2020). Comparison of different uncoated and starch-coated superparamagnetic iron oxide nanoparticles: implications for stem cell tracking. *Int. J. Biol. Macromol.* 143 763–774. 10.1016/j.ijbiomac.2019.10.031 31626822

[B48] Ferreira L. J. (2009). Nanoparticles as tools to study and control stem cells. *J. Cell Biochem.* 108 746–752. 10.1002/jcb.22303 19708027

[B49] Friedenstein A. Chailakhjan R. Lalykina K. (1970). The development of fibroblast colonies in monolayer cultures of guinea-pig bone marrow and spleen cells. *Cell Prolif.* 3 393–403. 10.1111/j.1365-2184.1970.tb00347.x 5523063

[B50] Fu Y. Azene N. Xu Y. Kraitchman D. L. (2011). Tracking stem cells for cardiovascular applications in vivo: focus on imaging techniques. *Imag. Med.* 3 473–486. 10.2217/iim.11.33 22287982 PMC3265127

[B51] Gao M. Chen J. Lin G. Li S. Wang L. Qin A. (2016). Long-term tracking of the osteogenic differentiation of mouse BMSCs by aggregation-induced emission nanoparticles. *ACS Appl. Mater.* 8 17878–17884. 10.1021/acsami.6b05471 27400339

[B52] Gao Y. Cui Y. Chan J. K. Xu C. J. (2013). Stem cell tracking with optically active nanoparticles. *Am. J. Nucl. Med. Mol. Imag.* 3 232–246. PMC3627520 23638335

[B53] Giljohann D. A. Seferos D. S. Daniel W. L. Massich M. D. Patel P. C. Mirkin C. A. (2010). Gold nanoparticles for biology and medicine. *Angew Chem. Int. Ed Engl.* 49 3280–3294. 20401880 10.1002/anie.200904359 PMC3930332

[B54] Gliga A. R. Skoglund S. Wallinder I. O. Fadeel B. Karlsson H. L. (2014). Size-dependent cytotoxicity of silver nanoparticles in human lung cells: the role of cellular uptake, agglomeration and Ag release. *Part Fibre Toxicol.* 11:11. 10.1186/1743-8977-11-11 24529161 PMC3933429

[B55] Godin B. Sakamoto J. H. Serda R. E. Grattoni A. Bouamrani A. Ferrari M. J. (2010). Emerging applications of nanomedicine for the diagnosis and treatment of cardiovascular diseases. *Trends Pharmacol. Sci.* 31 199–205. 10.1016/j.tips.2010.01.003 20172613 PMC2862836

[B56] Goradel N. H. Hour F. G. Negahdari B. Malekshahi Z. V. Hashemzehi M. Masoudifar A. (2018). Stem cell therapy: a new therapeutic option for cardiovascular diseases. *J. Cell Biochem.* 119 95–104. 10.1002/jcb.26169 28543595

[B57] Grauss R. W. Winter E. M. van Tuyn J. Pijnappels D. A. Steijn R. V. Hogers B. (2007). Mesenchymal stem cells from ischemic heart disease patients improve left ventricular function after acute myocardial infarction. *Am. J. Physiol. Heart Circ. Physiol.* 293 H2438–H2447. 17644573 10.1152/ajpheart.00365.2007

[B58] Gu L. Li X. Jiang J. Guo G. Wu H. Wu M. (2018). Stem cell tracking using effective self-assembled peptide-modified superparamagnetic nanoparticles. *Nanoscale* 10 15967–15979. 10.1039/c7nr07618e 29916501

[B59] Guldris N. Argibay B. R. Gallo J. Iglesias-Rey R. N. Carbo-Argibay E. Kolen’ko Y. V. (2017). Magnetite nanoparticles for stem cell labeling with high efficiency and long-term in vivo tracking. *Bioconjugate Chem.* 28 362–370. 10.1021/acs.bioconjchem.6b00522 27977143

[B60] Guo B. Chen J. Chen N. Middha E. Xu S. Pan Y. (2019a). High-Resolution 3D NIR-II photoacoustic imaging of cerebral and tumor vasculatures using conjugated polymer nanoparticles as contrast agent. *Adv. Mater.* 31:1808355. 10.1002/adma.201808355 31063244

[B61] Guo B. Feng Z. Hu D. Xu S. Middha E. Pan Y. (2019b). Precise deciphering of brain vasculatures and microscopic tumors with dual NIR-II fluorescence and photoacoustic imaging. *Adv. Mater.* 31:1902504. 10.1002/adma.201902504 31169334

[B62] Guo Y. Yu Y. Hu S. Chen Y. Shen Z. J. (2020). The therapeutic potential of mesenchymal stem cells for cardiovascular diseases. *J. Mol. Endocrinol.* 11 R109–R120. 10.1038/s41419-020-2542-9 PMC7214402 32393744

[B63] Hachani R. Birchall M. A. Lowdell M. W. Kasparis G. Tung L. D. Manshian B. B. (2017). Assessing cell-nanoparticle interactions by high content imaging of biocompatible iron oxide nanoparticles as potential contrast agents for magnetic resonance imaging. *Sci. Rep.* 7:7850. 10.1038/s41598-017-08092-w PMC5552868 28798327

[B64] Hahn M. A. Singh A. K. Sharma P. Brown S. C. Moudgil B. M. (2011). Nanoparticles as contrast agents for in-vivo bioimaging: current status and future perspectives. *Anal. Bioanal. Chem.* 399 3–27. 10.1007/s00216-010-4207-5 20924568

[B65] Hao J. Zhu W. Sheng C. Yu Y. Zhou Q. J. (2009). Human parthenogenetic embryonic stem cells: one potential resource for cell therapy. *Sci. China C Life Sci.* 52 599–602. 10.1007/s11427-009-0096-2 19641863

[B66] Hao L. Zou Z. Tian H. Zhang Y. Zhou H. Liu L. J. (2014). Stem cell-based therapies for ischemic stroke. *Biomed. Res. Int.* 2014:468748. 10.1155/2014/468748 PMC3955655 24719869

[B67] Herschman H. R. (2004). PET reporter genes for noninvasive imaging of gene therapy, cell tracking and transgenic analysis. *Crit. Rev. Oncol. Hematol.* 51 191–204. 10.1016/j.critrevonc.2004.04.006 15331078

[B68] Heslop J. A. Hammond T. G. Santeramo I. Tort Piella A. Hopp I. Zhou J. (2015). Concise review: workshop review: understanding and assessing the risks of stem cell-based therapies. *Stem Cells Transl. Med.* 4 389–400. 25722427 10.5966/sctm.2014-0110 PMC4367503

[B69] Hong H. Yang Y. Zhang Y. Cai W. J. (2010). Non-invasive cell tracking in cancer and cancer therapy. *Curr. Top Med. Chem.* 10 1237–1248. 10.2174/156802610791384234 20388105 PMC2916057

[B70] Hsiao J. K. Tsai C. P. Chung T. H. Hung Y. Yao M. Liu H. M. (2008). Mesoporous silica nanoparticles as a delivery system of gadolinium for effective human stem cell tracking. *Small* 4 1445–1452. 10.1002/smll.200701316 18680095

[B71] Hsieh S.-C. Wang F.-F. Lin C.-S. Chen Y.-J. Hung S.-C. Wang Y.-J. (2006). The inhibition of osteogenesis with human bone marrow mesenchymal stem cells by CdSe/ZnS quantum dot labels. *Biomaterials* 27 1656–1664. 10.1016/j.biomaterials.2005.09.004 16188313

[B72] Huang D. M. Hung Y. Ko B. S. Hsu S. C. Chen W. H. Chien C. L. (2005). Highly efficient cellular labeling of mesoporous nanoparticles in human mesenchymal stem cells: implication for stem cell tracking. *FASEB J.* 19 2014–2016. 10.1096/fj.05-4288fje 16230334

[B73] Huang J. Ning X. Luo W. Chen M. Wang Z. Zhang W. (2020). CT/NIRF dual-modal imaging tracking and therapeutic efficacy of transplanted mesenchymal stem cells labeled with Au nanoparticles in silica-induced pulmonary fibrosis. *J. Mater. Chem. B* 8 1713–1727. 10.1039/c9tb02652e 32022096

[B74] Idris N. M. Li Z. Ye L. Sim E. K. W. Mahendran R. Ho P. C.-L. (2009). Tracking transplanted cells in live animal using upconversion fluorescent nanoparticles. *Biomaterials* 30 5104–5113. 10.1016/j.biomaterials.2009.05.062 19539368

[B75] Jaiswal J. K. Goldman E. R. Mattoussi H. Simon S. M. (2004). Use of quantum dots for live cell imaging. *Nat. Methods* 1 73–78. 10.1038/nmeth1004-73 16138413

[B76] Jaiswal S. Jamieson C. H. Pang W. W. Park C. Y. Chao M. P. Majeti R. (2009). CD47 is upregulated on circulating hematopoietic stem cells and leukemia cells to avoid phagocytosis. *Cell* 138 271–285. 10.1016/j.cell.2009.05.046 19632178 PMC2775564

[B77] Jambhrunkar S. Qu Z. Popat A. Yang J. Noonan O. Acauan L. (2014). Effect of surface functionality of silica nanoparticles on cellular uptake and cytotoxicity. *Mol. Pharm.* 11 3642–3655. 10.1021/mp500385n 25166282

[B78] Jiang R. Liao Y. Yang F. Cheng Y. Dai X. Chao J. J. (2019). SPIO nanoparticle-labeled bone marrow mesenchymal stem cells inhibit pulmonary EndoMT induced by SiO2. *Exp. Cell Res.* 383:111492. 10.1016/j.yexcr.2019.07.005 31291564

[B79] Jing X.-H. Yang L. Duan X.-J. Xie B. Chen W. Li Z. (2008). In vivo MR imaging tracking of magnetic iron oxide nanoparticle labeled, engineered, autologous bone marrow mesenchymal stem cells following intra-articular injection. *Joint Bone Spine* 75 432–438. 10.1016/j.jbspin.2007.09.013 18448377

[B80] Kang H. Zhang K. Pan Q. Lin S. Wong D. S. H. Li J. (2018). Remote control of intracellular calcium using upconversion nanotransducers regulates stem cell differentiation in vivo. *Adv. Funct. Mater.* 28:1802642. 10.1002/adfm.201802642

[B81] Kastrup J. J. (2011). Stem cells therapy for cardiovascular repair in ischemic heart disease: how to predict and secure optimal outcome? *EPMA J.* 2 107–117. 10.1007/s13167-011-0062-5 23199132 PMC3405371

[B82] Khan I. Saeed K. Khan I. J. (2019). Nanoparticles: Properties, applications and toxicities. *Arabian J. Chem.* 12 908–931. 10.1016/j.arabjc.2017.05.011

[B83] Kim H. Dae H.-M. Park C. Kim E. O. Kim D. Kim I.-H. (2011). A highly sensitive magnetite nanoparticle as a simple and rapid stem cell labelling agent for MRI tracking. *J. Mater. Chem.* 21 7742–7747. 10.1039/c1jm10247h

[B84] Kim J. Shapiro L. Flynn A. J. (2015a). The clinical application of mesenchymal stem cells and cardiac stem cells as a therapy for cardiovascular disease. *Pharmacol Ther.* 151 8–15. 10.1016/j.pharmthera.2015.02.003 25709098

[B85] Kim K. S. Park W. Na K. J. B. (2015b). Gadolinium-chelate nanoparticle entrapped human mesenchymal stem cell via photochemical internalization for cancer diagnosis. *Biomaterials* 36 90–97. 10.1016/j.biomaterials.2014.09.014 25301637

[B86] Kim M. H. Lee Y. J. Kang J. H. (2016a). Stem cell monitoring with a direct or indirect labeling method. *Nucl. Med. Mol. Imag.* 50 275–283. 10.1007/s13139-015-0380-y 27994682 PMC5135688

[B87] Kim S. J. Lewis B. Steiner M. S. Bissa U. V. Dose C. Frank J. A. (2016b). Superparamagnetic iron oxide nanoparticles for direct labeling of stem cells and in vivo MRI tracking. *Contrast Media Mol. Imag.* 11 55–64. 10.1002/cmmi.1658 26234504 PMC4729653

[B88] Kircher M. F. Gambhir S. S. Grimm J. J. (2011). Noninvasive cell-tracking methods. *Nat. Rev. Clin. Oncol.* 8:677. 10.1038/nrclinonc.2011.141 21946842

[B89] Kraitchman D. L. Bulte J. W. J. A. (2009). In vivo imaging of stem cells and beta cells using direct cell labeling and reporter gene methods. *Arterioscler Thromb Vasc. Biol.* 29 1025–1030. 10.1161/atvbaha.108.165571 19359666 PMC2866294

[B90] Krebsbach P. H. Robey P. G. (2002). Dental and skeletal stem cells: potential cellular therapeutics for craniofacial regeneration. *J. Dent Educ.* 66 766–773. 10.1002/j.0022-0337.2002.66.6.tb03557.x 12117099

[B91] Laffey M. K. Kubelick K. P. Donnelly E. M. Emelianov S. Y. (2020). Effects of freezing on mesenchymal stem cells labeled with gold nanoparticles. *Tissue Eng. Part C Methods* 26 1–10. 10.1089/ten.tec.2019.0198 31724492

[B92] Lankoff A. Arabski M. Wegierek-Ciuk A. Kruszewski M. Lisowska H. Banasik-Nowak A. (2012). Effect of surface modification of silica nanoparticles on toxicity and cellular uptake by human peripheral blood lymphocytes in vitro. *Nanotoxicology* 7 235–250. 10.3109/17435390.2011.649796 22264124

[B93] Lee H. Park I. Kim H. Kim S. J. (2009). Human neural stem cells overexpressing glial cell line-derived neurotrophic factor in experimental cerebral hemorrhage. *Gene Ther.* 16 1066–1076. 10.1038/gt.2009.51 19554035

[B94] Lee S. H. Park D. J. Yun W. S. Park J.-E. Choi J. S. Key J. (2020). Endocytic trafficking of polymeric clustered superparamagnetic iron oxide nanoparticles in mesenchymal stem cells. *J. Control. Release* 326 408–418. 10.1016/j.jconrel.2020.07.032 32711024

[B95] Lee S.-T. Chu K. Park J.-E. Lee K. Kang L. Kim S. U. (2005). Intravenous administration of human neural stem cells induces functional recovery in Huntington’s disease rat model. *Neurosci. Res.* 52 243–249. 10.1016/j.neures.2005.03.016 15896865

[B96] Lewandowska-Łańcucka J. Staszewska M. Szuwarzyński M. Kêpczyński M. Romek M. Tokarz W. (2014). Synthesis and characterization of the superparamagnetic iron oxide nanoparticles modified with cationic chitosan and coated with silica shell. *J. Alloys Compounds* 586 45–51. 10.1016/j.jallcom.2013.10.039

[B97] Lewin M. Carlesso N. Tung C.-H. Tang X.-W. Cory D. Scadden D. T. (2000). Tat peptide-derivatized magnetic nanoparticles allow in vivo tracking and recovery of progenitor cells. *Nat. Biotechnol.* 18 410–414. 10.1038/74464 10748521

[B98] Li J. Lee W. Y. Wu T. Xu J. Zhang K. Li G. (2016). Multifunctional quantum dot nanoparticles for effective differentiation and long-term tracking of human mesenchymal stem cells in vitro and in vivo. *Adv. Healthc. Mater.* 5 1049–1057. 10.1002/adhm.201500879 26919348

[B99] Li L. Jiang W. Luo K. Song H. Lan F. Wu Y. (2013). Superparamagnetic iron oxide nanoparticles as MRI contrast agents for non-invasive stem cell labeling and tracking. *Theranostics* 3 595–615. 10.7150/thno.5366 23946825 PMC3741608

[B100] Li Y. He J. Wang F. Ju Z. Liu S. Zhang Y. (2010). Differentiation of embryonic stem cells in adult bone marrow. *J. Genet. Genomics* 37 431–439. 20659707 10.1016/S1673-8527(09)60062-X

[B101] Li Y. Song Y. Zhao L. Gaidosh G. Laties A. M. Wen R. J. (2008). Direct labeling and visualization of blood vessels with lipophilic carbocyanine dye DiI. *Nat. Protoc.* 3 1703–1708. 10.1038/nprot.2008.172 18846097 PMC2811090

[B102] Liang C. Wang C. Liu Z. J. P. (2013). Stem cell labeling and tracking with nanoparticles. *Stem Cells Int.* 30 1006–1017.

[B103] Liao N. Wu M. Pan F. Lin J. Li Z. Zhang D. (2016). Poly (dopamine) coated superparamagnetic iron oxide nanocluster for noninvasive labeling, tracking, and targeted delivery of adipose tissue-derived stem cells. *Sci. Rep.* 6:18746. 10.1038/srep18746 PMC4700528 26728448

[B104] Lilly M. A. Davis M. F. Fabie J. E. Terhune E. B. Gallicano G. I. (2016). Current stem cell based therapies in diabetes. *Am. J. Stem Cells* 5 87–98. 27853630 PMC5107653

[B105] Lim S. Yoon H. Y. Jang H. J. Song S. Kim W. Park J. (2019). Dual-modal imaging-guided precise tracking of bioorthogonally labeled mesenchymal stem cells in mouse brain stroke. *ACS Nano* 13 10991–11007. 10.1021/acsnano.9b02173 31584257

[B106] Lin W. (2015). *Introduction: Nanoparticles in Medicine.* Washington, DC: ACS Publications. 10.1021/acs.chemrev.5b00534 26463639

[B107] Liu G. Wang Z. Lu J. Xia C. Gao F. Gong Q. (2011). Low molecular weight alkyl-polycation wrapped magnetite nanoparticle clusters as MRI probes for stem cell labeling and in vivo imaging. *Biomaterials* 32 528–537. 10.1016/j.biomaterials.2010.08.099 20869767

[B108] Loewen N. Fautsch M. P. Teo W.-L. Bahler C. K. Johnson D. H. Poeschla E. M. (2004). Long-term, targeted genetic modification of the aqueous humor outflow tract coupled with noninvasive imaging of gene expression in vivo. *Invest. Ophthalmol. Vis. Sci.* 45 3091–3098. 10.1167/iovs.04-0366 15326125

[B109] Lu C.-W. Hung Y. Hsiao J.-K. Yao M. Chung T.-H. Lin Y.-S. (2007). Bifunctional magnetic silica nanoparticles for highly efficient human stem cell labeling. *Nano Lett.* 7 149–154. 10.1021/nl0624263 17212455

[B110] Lusic H. Grinstaff M. W. (2013). X-ray-computed tomography contrast agents. *Chem. Rev.* 113 1641–1666. 10.1021/cr200358s 23210836 PMC3878741

[B111] Lv F.-J. Tuan R. S. Cheung K. M. Leung V. Y. (2014). Concise review: the surface markers and identity of human mesenchymal stem cells. *Stem Cells* 32 1408–1419. 10.1002/stem.1681 24578244

[B112] Ma Y. Ji Y. You M. Wang S. Dong Y. Jin G. (2016). Labeling and long-term tracking of bone marrow mesenchymal stem cells in vitro using NaYF4: Yb3+, Er3+ upconversion nanoparticles. *Acta Biomater.* 42 199–208. 10.1016/j.actbio.2016.07.030 27435964

[B113] Mahmoudi M. Sant S. Wang B. Laurent S. Sen T. J. (2011). Superparamagnetic iron oxide nanoparticles (SPIONs): development, surface modification and applications in chemotherapy. *Adv. Drug Delivery Rev.* 63 24–46. 10.1016/j.addr.2010.05.006 20685224

[B114] Maumus M. Guérit D. Toupet K. Jorgensen C. Noël D. J. (2011). Mesenchymal stem cell-based therapies in regenerative medicine: applications in rheumatology. *Stem Cells Res. Ther.* 2:14. 10.1186/scrt55 21457518 PMC3226285

[B115] Mazumder S. Dey R. Mitra M. Mukherjee S. Das G. J. (2009). Biofunctionalized quantum dots in biology and medicine. *J. Nanomaterials* 2009:815734.

[B116] McNamara K. Tofail S. A. (2017). Nanoparticles in biomedical applications. *Adv. Phys.* 2 54–88. 10.1039/c5cp00831j 26024211

[B117] Meir R. Popovtzer R. J. (2018). Cell tracking using gold nanoparticles and computed tomography imaging. Wiley Interdiscip. *Rev. Nanomed. Nanobiotechnol.* 10:e1480. 10.1002/wnan.1480 28544497

[B118] Miguel O. B. Gossuin Y. Morales M. Gillis P. Muller R. Veintemillas-Verdaguer S. J. (2007). Comparative analysis of the 1H NMR relaxation enhancement produced by iron oxide and core-shell iron–iron oxide nanoparticles. *Magn. Reson. Imaging* 25 1437–1441. 10.1016/j.mri.2007.04.006 17566686

[B119] Mikkola H. K. Orkin S. H. J. D. (2006). The journey of developing hematopoietic stem cells. *Development* 133 3733–3744. 10.1242/dev.02568 16968814

[B120] Murphy C. J. Gole A. M. Stone J. W. Sisco P. N. Alkilany A. M. Goldsmith E. C. (2008). Gold nanoparticles in biology: beyond toxicity to cellular imaging. *Acc. Chem. Res.* 41 1721–1730. 10.1021/ar800035u 18712884

[B121] Na H. B. Hyeon T. J. (2009). Nanostructured T1 MRI contrast agents. *J. Mater. Chem.* 19 6267–6273. 10.1039/b902685a

[B122] Nafiujjaman M. Kim T. (2020). Gold nanoparticles as a computed tomography marker for stem cell tracking. *Methods Mol. Biol.* 2126 155–166. 10.1007/978-1-0716-0364-2_14 32112387

[B123] Ni Z. Chen R. (2015). Transcranial magnetic stimulation to understand pathophysiology and as potential treatment for neurodegenerative diseases. *Transl. Neurodegener.* 4:22. 10.1186/s40035-015-0045-x 26579223 PMC4647804

[B124] Park W. Yang H. N. Ling D. Yim H. Kim K. S. Hyeon T. (2014). Multi-modal transfection agent based on monodisperse magnetic nanoparticles for stem cell gene delivery and tracking. *Biomaterials* 35 7239–7247. 10.1016/j.biomaterials.2014.05.010 24881029

[B125] Patrick P. S. Kolluri K. K. Thin M. Z. Edwards A. Sage E. K. Sanderson T. (2020). Lung delivery of MSCs expressing anti-cancer protein TRAIL visualised with 89 Zr-oxine PET-CT. *Stem Cell Res. Ther.* 11:256. 10.1186/s13287-020-01770-z PMC7318529 32586403

[B126] Peng X. Li C. Bai Y. Wang X. Zhang Y. An Y. (2018). Noninvasive evaluation of the migration effect of transplanted endothelial progenitor cells in ischemic muscle using a multimodal imaging agent. *Int. J. Nanomed.* 13 1819–1829. 10.2147/ijn.s152976 29606873 PMC5868615

[B127] Perez J. R. Ybarra N. Chagnon F. Serban M. Lee S. Seuntjens J. (2017). Tracking of mesenchymal stem cells with fluorescence endomicroscopy imaging in radiotherapy-induced lung injury. *Sci. Rep.* 7:40748. 10.1038/srep40748 PMC5244404 28102237

[B128] Ratzinger G. Agrawal P. Körner W. Lonkai J. Sanders H. M. Terreno E. (2010). Surface modification of PLGA nanospheres with Gd-DTPA and Gd-DOTA for high-relaxivity MRI contrast agents. *Biomaterials* 31 8716–8723. 10.1016/j.biomaterials.2010.07.095 20797782

[B129] Rawat S. Gupta S. Bhat M. Dinda A. K. Mohanty S. (2019). Efficient labeling of human mesenchymal stem cells using iron oxide nanoparticles. *Methods Mol. Biol.* 2150 113–120. 10.1007/7651_2019_265 31707646

[B130] Ren N. Liang N. Yu X. Wang A. Xie J. Sun C. J. N. (2020). Ligand-free upconversion nanoparticles for cell labeling and their effects on stem cell differentiation. *Nanotechnology* 31:145101. 10.1088/1361-6528/ab62cc 31846954

[B131] Rhyner M. N. Smith A. M. Gao X. Mao H. Yang L. Nie S. (2006). Quantum dots and multifunctional nanoparticles: new contrast agents for tumor imaging. *Nanomedicine* 1 209–217. 10.2217/17435889.1.2.209 17716110

[B132] Riera R. Feiner-Gracia N. Fornaguera C. Cascante A. Borrós S. Albertazzi L. J. N. (2019). Tracking the DNA complexation state of PBAE polyplexes in cells with super resolution microscopy. *Nanoscale* 11 17869–17877. 10.1039/c9nr02858g 31552987

[B133] Robert P. Lehericy S. Grand S. Violas X. Fretellier N. Idée J.-M. (2015). T1-weighted hypersignal in the deep cerebellar nuclei after repeated administrations of gadolinium-based contrast agents in healthy rats: difference between linear and macrocyclic agents. *Invest. Radiol.* 50 473–480. 10.1097/rli.0000000000000181 26107651 PMC4494686

[B134] Saha K. Kim S. T. Yan B. Miranda O. R. Alfonso F. S. Shlosman D. (2013). Surface functionality of nanoparticles determines cellular uptake mechanisms in mammalian cells. *Small* 9 300–305. 10.1002/smll.201201129 22972519 PMC4070423

[B135] Santelli J. Lechevallier S. Baaziz H. Vincent M. Martinez C. Mauricot R. (2018). Multimodal gadolinium oxysulfide nanoparticles: a versatile contrast agent for mesenchymal stem cell labeling. *Nanoscale* 10 16775–16786. 10.1039/c8nr03263g 30156241

[B136] Santoso M. R. Yang P. C. (2016). Magnetic nanoparticles for targeting and imaging of stem cells in myocardial infarction. *Stem Cells Int.* 2016:4198790. 10.1155/2016/4198790 PMC4834159 27127519

[B137] Schmitz N. Pfistner B. Sextro M. Sieber M. Carella A. M. Haenel M. (2002). Aggressive conventional chemotherapy compared with high-dose chemotherapy with autologous haemopoietic stem-cell transplantation for relapsed chemosensitive Hodgkin’s disease: a randomised trial. *Lancet* 359 2065–2071. 10.1016/S0140-6736(02)08938-9 12086759

[B138] Sehl O. C. Makela A. V. Hamilton A. M. Foster P. J. (2019). Trimodal cell tracking in vivo: combining iron-and fluorine-based magnetic resonance imaging with magnetic particle imaging to monitor the delivery of mesenchymal stem cells and the ensuing inflammation. *Tomography* 5 367–376. 10.18383/j.tom.2019.00020 31893235 PMC6935990

[B139] Seleverstov O. Zabirnyk O. Zscharnack M. Bulavina L. Nowicki M. Heinrich J.-M. (2006). Quantum dots for human mesenchymal stem cells labeling. a size-dependent autophagy activation. *Nano Lett.* 6 2826–2832. 10.1021/nl0619711 17163713

[B140] Shah B. S. Clark P. A. Moioli E. K. Stroscio M. A. Mao J. J. (2007). Labeling of mesenchymal stem cells by bioconjugated quantum dots. *Nano Lett.* 7 3071–3079. 10.1021/nl071547f 17887799 PMC4410692

[B141] Shang Y. Li T. Yu G. J. (2017). Clinical applications of near-infrared diffuse correlation spectroscopy and tomography for tissue blood flow monitoring and imaging. *Physiol. Meas.* 38 R1–R26. 10.1088/1361-6579/aa60b7 28199219 PMC5726862

[B142] Sherry A. D. Cacheris W. P. Kuan K. T. (1988). Stability constants for Gd3+ binding to model DTPA-conjugates and DTPA-proteins: implications for their use as magnetic resonance contrast agents. *Magn. Reson. Med.* 8 180–190. 10.1002/mrm.1910080208 3210955

[B143] Shin T. H. Lee D. Y. Ketebo A. A. Lee S. Manavalan B. Basith S. (2019). Silica-coated magnetic nanoparticles decrease human bone marrow-derived mesenchymal stem cell migratory activity by reducing membrane fluidity and impairing focal adhesion. *Nanomaterials* 9:1475. 10.3390/nano9101475 31627375 PMC6835988

[B144] Singh N. Jenkins G. J. Asadi R. Doak S. H. (2010). Potential toxicity of superparamagnetic iron oxide nanoparticles (SPION). *Nano Rev.* 1:5358. 10.3402/nano.v1i0.5358 22110864 PMC3215220

[B145] Srivastava D. Ivey K. N. (2006). Potential of stem-cell-based therapies for heart disease. *Nature* 441 1097–1099. 10.1038/nature04961 16810246

[B146] Stenudd M. Sabelström H. Frisén J. J. (2015). Role of endogenous neural stem cells in spinal cord injury and repair. *JAMA Neurol.* 72 235–237. 10.1001/jamaneurol.2014.2927 25531583

[B147] Su X. Shen Y. Weintraub N. L. Tang Y. (2019). Imaging and tracking stem cell engraftment in ischemic hearts by near-infrared fluorescent protein (iRFP) labeling. *Methods Mol. Biol.* 2150 121–129. 10.1007/7651_2019_226 PMC7082122 31020637

[B148] Sutton E. J. Henning T. D. Pichler B. J. Bremer C. Daldrup-Link H. E. (2008). Cell tracking with optical imaging. *Eur. Radiol.* 18 2021–2032. 10.1007/s00330-008-0984-z 18506449

[B149] Szpak A. Kania G. Skórka T. Tokarz W. Zapotoczny S. Nowakowska M. J. (2013). Stable aqueous dispersion of superparamagnetic iron oxide nanoparticles protected by charged chitosan derivatives. *J. Nanopart. Res.* 15:1372. 10.1007/s11051-012-1372-9 23420339 PMC3568472

[B150] Temple S. J. (2001). The development of neural stem cells. *Nature* 414 112–117. 10.1038/35102174 11689956

[B151] Tkaczyk E. R. (2017). Innovations and developments in dermatologic non-invasive optical imaging and potential clinical applications. *Acta Derm. Venereol.* 128 5–13. 10.2340/00015555-2717 28676880 PMC5943168

[B152] Trivedi P. Tray N. Nguyen T. Nigam N. Gallicano G. I. (2010). Mesenchymal stem cell therapy for treatment of cardiovascular disease: helping people sooner or later. *Stem Cells Dev.* 19 1109–1120. 10.1089/scd.2009.0465 20092388

[B153] Tseng C.-L. Shih I.-L. Stobinski L. Lin F.-H. (2010). Gadolinium hexanedione nanoparticles for stem cell labeling and tracking via magnetic resonance imaging. *Biomaterials* 31 5427–5435. 10.1016/j.biomaterials.2010.03.049 20400176

[B154] Turjeman K. Bavli Y. Kizelsztein P. Schilt Y. Allon N. Katzir T. B. (2015). Nano-Drugs based on nano sterically stabilized liposomes for the treatment of inflammatory neurodegenerative diseases. *PLoS One* 10:e0130442. 10.1371/journal.pone.0130442 26147975 PMC4492950

[B155] Uccelli A. Moretta L. Pistoia V. J. (2008). Mesenchymal stem cells in health and disease. *Nat. Rev. Immunol.* 8 726–736. 10.1038/nri2395 19172693

[B156] Vagnozzi R. J. Maillet M. Sargent M. A. Khalil H. Johansen A. K. Z. Schwanekamp J. A. (2020). An acute immune response underlies the benefit of cardiac stem cell therapy. *Nature* 577 405–409. 10.1038/s41586-019-1802-2 31775156 PMC6962570

[B157] Van Den Bos E. J. Wagner A. Mahrholdt H. Thompson R. B. Morimoto Y. Sutton B. S. (2003). Improved efficacy of stem cell labeling for magnetic resonance imaging studies by the use of cationic liposomes. *Cell Transplant.* 12 743–756. 10.3727/000000003108747352 14653621

[B158] Walczak P. Bulte J. W. (2007). The role of noninvasive cellular imaging in developing cell-based therapies for neurodegenerative disorders. *Neurodegener. Dis.* 4 306–313. 10.1159/000101887 17627134

[B159] Wang C. Cheng L. Xu H. Liu Z. J. B. (2012a). Towards whole-body imaging at the single cell level using ultra-sensitive stem cell labeling with oligo-arginine modified upconversion nanoparticles. *Biomaterials* 33 4872–4881. 10.1016/j.biomaterials.2012.03.047 22483011

[B160] Wang C. Ma X. Ye S. Cheng L. Yang K. Guo L. (2012b). Protamine functionalized single-walled carbon nanotubes for stem cell labeling and in vivo raman/magnetic resonance/photoacoustic triple-modal imaging. *Adv. Funct. Mater.* 22 2363–2375. 10.1002/adfm.201200133

[B161] Wang F. Banerjee D. Liu Y. Chen X. Liu X. J. A. (2010). Upconversion nanoparticles in biological labeling, imaging, and therapy. *Analyst* 135 1839–1854. 10.1039/c0an00144a 20485777

[B162] Wang Y. Xu C. Ow H. J. T. (2013). Commercial nanoparticles for stem cell labeling and tracking. *Theranostics* 3:544. 10.7150/thno.5634 23946821 PMC3741604

[B163] Wu C. Li J. Pang P. Liu J. Zhu K. Li D. (2014). Polymeric vector-mediated gene transfection of MSCs for dual bioluminescent and MRI tracking in vivo. *Biomaterials* 35 8249–8260. 10.1016/j.biomaterials.2014.06.014 24976241

[B164] Wu Y. Chen L. Scott P. G. Tredget E. E. (2007). Mesenchymal stem cells enhance wound healing through differentiation and angiogenesis. *Stem Cells* 25 2648–2659. 10.1634/stemcells.2007-0226 17615264

[B165] Xie B. Gu P. Wang W. Dong C. Zhang L. Zhang J. (2016). Therapeutic effects of human umbilical cord mesenchymal stem cells transplantation on hypoxic ischemic encephalopathy. *Am. J. Transl. Res.* 8:3241. PMC4969462 27508046

[B166] Xie Y. Liu W. Zhang B. Wang B. Wang L. Liu S. (2019). Systematic intracellular biocompatibility assessments of superparamagnetic iron oxide nanoparticles in human umbilical cord mesenchyme stem cells in testifying its reusability for inner cell tracking by MRI. *J. Biomed. Nanotechnol.* 15 2179–2192. 10.1166/jbn.2019.2845 31847932

[B167] Yao M. Shi X. Zuo C. Ma M. Zhang L. Zhang H. (2020). Engineering of SPECT/Photoacoustic imaging/antioxidative stress triple-function nanoprobe for advanced mesenchymal stem cell therapy of cerebral ischemia. *ACS Appl. Mater. Interfaces* 12 37885–37895. 10.1021/acsami.0c10500 32806884

[B168] Ye L. Chang Y.-H. Xiong Q. Zhang P. Zhang L. Somasundaram P. (2014). Cardiac repair in a porcine model of acute myocardial infarction with human induced pluripotent stem cell-derived cardiovascular cells. *Cell Stem Cell* 15 750–761. 10.1016/j.stem.2014.11.009 25479750 PMC4275050

[B169] Ye L. Haider H. K. Sim E. K. (2006). Adult stem cells for cardiac repair: a choice between skeletal myoblasts and bone marrow stem cells. *Exp. Biol. Med.* 231 8–19. 10.1177/153537020623100102 16380640

[B170] Yi D. K. Nanda S. S. Kim K. Selvan S. T. (2017). Recent progress in nanotechnology for stem cell differentiation, labeling, tracking and therapy. *J. Mater. Chem. B* 5 9429–9451. 10.1039/C7TB02532G 32264559

[B171] Yin C. Wen G. Liu C. Yang B. Lin S. Huang J. (2018). Organic semiconducting polymer nanoparticles for photoacoustic labeling and tracking of stem cells in the second near-infrared window. *ACS Nano* 12 12201–12211. 10.1021/acsnano.8b05906 30433761

[B172] Young H. E. Black A. C. (2004). Adult stem cells. *Anat. Rec. A Discov. Mol. Cell Evol. Biol.* 276 75–102. 10.1002/ar.a.10134 14699636

[B173] Yukawa H. Baba Y. J. A. C. (2017). In vivo fluorescence imaging and the diagnosis of stem cells using quantum dots for regenerative medicine. *Anal. Chem.* 89 2671–2681. 10.1021/acs.analchem.6b04763 28194939

[B174] Zampelas A. Magriplis E. J. (2020). Dietary patterns and risk of cardiovascular diseases: a review of the evidence. *Proc. Nutr. Soc.* 79 68–75. 10.1017/S0029665119000946 31250769

[B175] Zhang L. Gu F. Chan J. Wang A. Langer R. Farokhzad O. J. (2008). Nanoparticles in medicine: therapeutic applications and developments. *Clin. Pharmacol. Ther.* 83 761–769. 10.1038/sj.clpt.6100400 17957183

[B176] Zhang S. J. Wu J. C. (2007). Comparison of imaging techniques for tracking cardiac stem cell therapy. *J. Nucl. Med.* 48 1916–1919. 10.2967/jnumed.107.043299 18056330 PMC3638042

[B177] Zhang Z. Hancock B. Leen S. Ramaswamy S. Sollott S. J. Boheler K. R. (2010). Compatibility of superparamagnetic iron oxide nanoparticle labeling for 1H MRI cell tracking with 31P MRS for bioenergetic measurements. *NMR Biomed.* 23 1166–1172. 10.1002/nbm.1545 20853523 PMC3161830

[B178] Zhao L. Kutikov A. Shen J. Duan C. Song J. Han G. J. T. (2013). Stem cell labeling using polyethylenimine conjugated (α-NaYbF4: Tm3+)/CaF2 upconversion nanoparticles. *Theranostics* 3 249–257. 10.7150/thno.5432 23606911 PMC3630525

[B179] Zheng Y. Huang J. Zhu T. Li R. Wang Z. Ma F. (2017). Stem cell tracking technologies for neurological regenerative medicine purposes. *Stem Cells Int.* 2017:2934149. 10.1155/2017/2934149 29138636 PMC5613625

[B180] Zhu D. Liu F. Ma L. Liu D. Wang Z. J. (2013). Nanoparticle-based systems for T1-weighted magnetic resonance imaging contrast agents. *Int. J. Mol. Sci.* 14 10591–10607. 10.3390/ijms140510591 23698781 PMC3676856

